# Methodologies for bacterial ribonuclease characterization using RNA-seq

**DOI:** 10.1093/femsre/fuad049

**Published:** 2023-09-01

**Authors:** Laura Broglia, Anaïs Le Rhun, Emmanuelle Charpentier

**Affiliations:** Max Planck Unit for the Science of Pathogens, D-10117 Berlin, Germany; Center for Human Technologies, Istituto Italiano di Tecnologia, 16152 Genova, Italy; Max Planck Unit for the Science of Pathogens, D-10117 Berlin, Germany; Univ. Bordeaux, CNRS, INSERM, ARNA, UMR 5320, U1212, F-33000 Bordeaux, France; Max Planck Unit for the Science of Pathogens, D-10117 Berlin, Germany; Institute for Biology, Humboldt University, D-10115 Berlin, Germany

**Keywords:** Ribonucleases, RNA sequencing, transcriptomics, post-transcriptional regulation, RNA processing, RNA decay, RNA degradation

## Abstract

Bacteria adjust gene expression at the post-transcriptional level through an intricate network of small regulatory RNAs and RNA-binding proteins, including ribonucleases (RNases). RNases play an essential role in RNA metabolism, regulating RNA stability, decay, and activation. These enzymes exhibit species-specific effects on gene expression, bacterial physiology, and different strategies of target recognition. Recent advances in high-throughput RNA sequencing (RNA-seq) approaches have provided a better understanding of the roles and modes of action of bacterial RNases. Global studies aiming to identify direct targets of RNases have highlighted the diversity of RNase activity and RNA-based mechanisms of gene expression regulation. Here, we review recent RNA-seq approaches used to study bacterial RNases, with a focus on the methods for identifying direct RNase targets.

## Teaser

This review presents and explores the different RNA sequencing strategies to investigate the activity of bacterial ribonucleases genome-wide.

## Introduction

Ribonucleases (RNases) are responsible for catalyzing the scission of the phosphodiester bond in RNA molecules. These enzymes are present in all living organisms, where they play a central role in RNA metabolism by cleaving RNA molecules internally (endoRNases) or from the extremities (exoRNases). Not only are they required for RNA turnover and subsequent nucleotide recycling, but they also participate in mechanisms of gene expression regulation.

**Figure 1.**
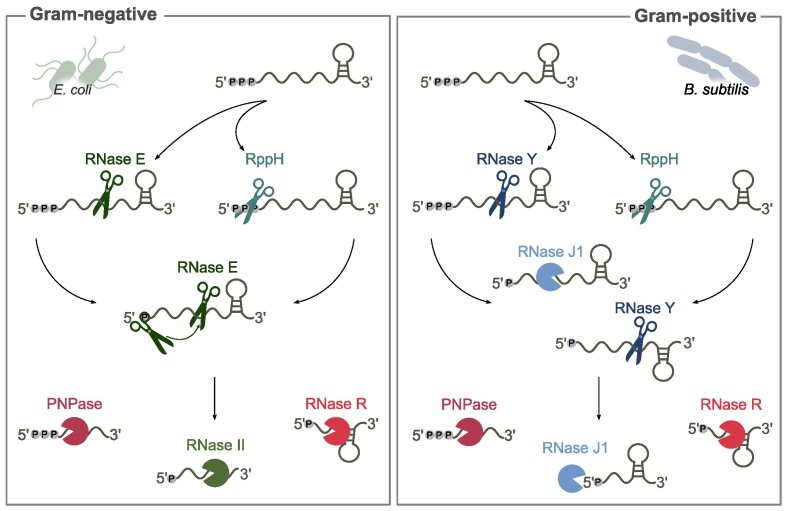
Bacterial RNA degradation pathways. Schematic representation of the *Escherichia coli* and *Bacillus subtilis* RNA degradation pathways. Degradation of RNA molecules is initiated by internal cleavage by an endoRNase⁠—RNase E or RNase Y⁠—represented as scissors. Alternatively, the 5′ triphosphate group (5′ PPP) is converted to 5′ monophosphate (5′ P) by a pyrophosphohydrolase (RppH). In *E. coli*, the 5′ P group stimulates RNase E activity, and target RNAs are cleaved multiple times by this RNase. In *B. subtilis*, the 5′ P RNA is targeted by either RNase Y or by RNase J1 (‘pacman’ symbol), which degrades the RNA from the 5′ to the 3′ end. The intermediate decay fragments are degraded by the 3′-to-5′ exoRNases (‘pacman’ symbols) RNase II (only in *E. coli*), PNPase and RNase R, or by RNase J1.

Although the set of RNases differs among bacterial species (Bechhofer and Deutscher 2019)—in particular between Gram-positive and Gram-negative bacteria—the main RNA degradation pathways are very similar (Fig. 1). RNA molecules are usually protected from RNase activity by a triphosphate group at the RNA 5′ terminus (5′ PPP) and by a terminator stem-loop at the 3′ terminus. Typically, RNA degradation is initiated either by the conversion of the 5′ PPP to 5′ monophosphate (5′ P) by a pyrophosphohydrolase (RppH) or by an internal cleavage within the RNA body catalyzed, for instance, by RNase E or RNase Y (Celesnik et al. 2007, 2007; Luciano et al. 2018). These events generate RNAs harboring 5′ P, which are further processed by endoRNases (*i.e*. RNase E and RNase Y) or a 5′-to-3′ exoRNase (*i.e*. RNase J1 mostly present in Gram-positive bacteria (Durand and Condon 2018)) that preferentially target 5′ P RNAs (Koslover et al. 2008, Richards et al. 2011, Laalami et al. 2014) (Fig. 1). The resulting decay intermediate fragments are rapidly degraded by 3′-to-5′ exoRNases, including PNPase and RNase R, or by RNase J1 (Fig. 1). Structured fragments (e.g. terminator stem-loops) are usually digested by RNase R, which displays an intrinsic helicase activity (Cheng and Deutscher 2005), or by PNPase acting in concert with RNA helicases (Liou et al. 2002, Chen et al. 2013). Furthermore, the repetitive addition of a poly(A) tail at the RNA 3′ ends, catalyzed by a poly(A) polymerase (PAP), generates RNA fragments more susceptible to 3′-to-5′ exoRNase degradation (O'Hara et al. 1995, Joanny et al. 2007). Indeed, these enzymes require a single-stranded RNA (ssRNA) overhang at the 3′ end of the substrate to bind the RNAs and further degrade them (Oussenko et al. 2005, Vincent and Deutscher 2006).

RNases play also a key role in RNA maturation, consisting of a processing event that results in the production of a functional RNA molecule. For example, ribosomal RNAs (rRNAs) and some transfer RNAs (tRNAs) are co-transcribed, and multiple endoribonucleolytic processing followed by trimming events are necessary for the production of the individual rRNA molecules (Deutscher 2006, 2009). Maturation events within polycistronic transcripts modulate the stability of the cleaved products, producing different stoichiometric amounts of proteins encoded in the same operon (Ludwig et al. 2001, Xu et al. 2015, DeLoughery et al. 2018).

RNases regulate gene expression at the post-transcriptional level by targeting specific mRNAs, leading to their degradation or stabilization. The targeting of a specific mRNA is achieved by modifying its accessibility to RNases, for instance, through the action of small noncoding RNAs (sRNAs) (Durand et al. 2015b, Carrier et al. 2018). Research over the past years have unveiled a plethora of sRNA/RNase mechanisms of gene expression regulation. sRNAs can directly affect the mRNA stability by recruiting RNases or by sequestering RNase cleavage sites on the bound mRNA (Ramirez-Peña et al. 2010, Durand et al. 2012). sRNAs can also control mRNA stability indirectly by promoting or inhibiting translation of the target mRNA (Durand et al. 2015a, Obana et al. 2017). The increase or decrease of ribosome density on the bound mRNA results in mRNA stabilization or degradation, respectively.

Finally, additional functions for bacterial RNases have been described. For instance, some RNases belong to toxin-antitoxin (TA) systems and act either as toxins by cleaving a wide range of RNAs under stress conditions and phage infection (Type II and III TAs), or as antitoxins by cleaving the mRNA encoding the toxin (Type V TA) (Cook et al. 2013, Masuda and Inouye 2017). Bacterial RNases also broadly participate in the modulation of Type I TA, consisting of an antisense RNA antitoxin that inhibits the production of its cognate toxin by base-pairing to its mRNA (Masachis and Darfeuille 2018). For instance, RNase III processing of the RNA duplexes formed by Type I TAs in *B. subtilis* is necessary for repression of the toxin expression, explaining why RNase III is essential in this bacterium (Durand et al. 2012, Reif et al. 2018). Furthermore, study of the clustered regularly interspaced short palindromic repeats (CRISPR)—CRISPR associated proteins (CRISPR-Cas) systems revealed an extensive involvement of RNases in several steps of the protection against invading foreign nucleic acids (Deltcheva et al. 2011, Chou-Zheng and Hatoum-Aslan 2019, Behler and Hess 2020).

Our current knowledge of bacterial RNase functions and mode of actions mostly stems from studies performed in the two model organisms *E. coli* and *B. subtilis*. However, investigation of the RNase activity in other bacteria revealed an enormous diversity among RNase orthologues in different bacterial species, suggesting that our understanding of RNA degradation and processing is far from complete. The investigation of the RNase properties and functions relies on the identification and characterization of their targets.

In the past, RNases have been studied largely *in vitro*, using biochemical approaches on a limited number of substrates. These studies are highly valuable, but they display several disadvantages. *In vitro* characterization of an RNase relies on the possibility to purify a tagged or untagged version of the RNase of interest, usually using a heterologous system. However, RNase purification can be considerably challenging, especially for membrane-associated RNases. *In vitro* assays offer the possibility to rapidly mutagenize the RNase under study and thereby pinpoint the key residues involved in the catalytic activity or in RNA binding. However, these approaches require prior knowledge of the target RNAs, which are commonly *in vitro* transcribed (IVT), and are limited by the number of target RNAs tested. In addition, IVT RNA substrates may form different secondary structures and lack RNA modifications when compared to the *in vivo* conditions. Likewise, recombinant RNase variants may lack post-translational modifications, which could affect the RNase processing. Finally, the absence of RNase interacting proteins and/or sRNAs, which are key factors in the control of RNase activity, in these experiments does not allow a complete recapitulation of the mechanism of RNase cleavage and may impair the mapping of RNase processing sites.

Comparing RNA abundance between a wild-type (WT) strain and a deletion mutant strain of the RNase of interest (e.g. Northern blot and primer extension analyses) is highly informative, but time-consuming and not high-throughput.

Transcriptomics approaches have become the method of choice for studying the roles of bacterial RNase, as they enable the simultaneous identification of multiple RNase targets and an understanding of the overall importance of the RNase under study. Transcriptomics studies, aimed at exploring the role and functions of bacterial RNases on a global scale, were pioneered using microarrays or tiling arrays. These techniques have made it possible to identify genes directly or indirectly regulated by an RNase and to monitor stability of mRNA transcripts in bacteria. In Supplementary Data 1, we have listed all the transcriptomic studies used to identify RNase targets. Microarrays-based studies have gradually been replaced by RNA sequencing (RNA-seq), which allows the simultaneous determination of RNA abundance and boundaries at the nucleotide resolution. This approach is particularly well suited to elucidating the impact of RNases on gene expression and determining the direct targets of RNases on a genome-wide scale. Over the past decade, researchers have established many RNA-seq-based methodologies to study bacterial RNases, providing large-scale data and improving our understanding of the mechanisms of action of bacterial RNases.

Here, we review the current RNA-seq methods for identifying RNase targets, discuss in detail the approaches for mapping cleavage positions and highlight the major advances in the field. Furthermore, we discuss the limitations of the available strategies and provide a perspective on how the study of bacterial RNases can benefit from the use of omics approaches.

## Bacterial RNA sequencing

In RNA-seq, a specific group of RNA molecules is usually converted into a library, composed of fragmented cDNA molecules harboring adapter sequences at both or one of the extremities. The cDNAs are amplified and subsequently sequenced at high throughput. Although multiple protocols for library preparation are available, some critical steps are common to all existing methods.

Regardless of the RNA species enriched, the majority of the protocols include steps for genomic DNA (gDNA) and rRNA removal. Exclusion of the gDNA by DNase treatment, ensures the reduction of background noise, while depletion of the rRNAs, which represent the most abundant RNA class present in the cell, allows more cDNAs nonderiving from rRNAs to be sequenced and therefore increases the information content of the sample.

Due to the limitation in cDNA length that can be sequenced by the majority of available RNA-seq platforms, the RNAs sometimes need to be fragmented. RNA fragmentation can be accomplished mechanically (e.g. acoustic shearing) (Creecy and Conway 2015), chemically (e.g. with divalent cations) (Sharma et al. 2010, Vivancos et al. 2010) or enzymatically (e.g. RNase III treatment) (Ares 2013). Alternatively, the fragmentation step could take place after the conversion of the RNAs to cDNAs.

Library preparation can be designated in order to preserve strand specificity, thereby allowing the identification of antisense RNAs (asRNAs) and the accurate mapping of bacterial transcripts (Levin et al. 2010). Strand-specificity is commonly maintained by differentiating the RNA 5′ and 3′ ends prior to cDNA synthesis. This can be achieved by addition of a poly(A) tail at the RNA 3′ ends followed by ligation of an 5′ adapter to the RNA (Sharma et al. 2010). Ligation of distinct adapters to the RNA 5′ and 3′ ends, respectively, can also be used as a method to retain strand specificity (Lu et al. 2007, Vivancos et al. 2010, Le Rhun et al. 2017). Alternatively, RNA can be converted to cDNA in the presence of actinomycin D, which inhibits the DNA-dependent DNA polymerase activity of the reverse transcriptase. Then, deoxy-UTP is used to synthesize the second-strand cDNA. Prior to amplification, the samples are treated with Uracil-N-Glycosylase to specifically degrade the strand containing uridine (Parkhomchuk et al. 2009, Lasa et al. 2011, Culviner and Laub 2018). Alternatively, a protocol, which excludes the synthesis of the second strand cDNA, was successfully applied to sequence single-stranded cDNA, providing information on both strands (Croucher et al. 2009).

To capture the information on the RNA ends and thus precisely map the 5′ or 3′ terminus, adapter sequences are often ligated to the RNAs prior to fragmentation. Ligation of adapters by RNA ligases requires the presence of specific chemical groups at the RNA extremities, such as 5′ P and 3′ hydroxyl (3′ OH). The pretreatment with enzymes that either modify the RNA ends or exclusively degrade RNAs harboring a specific chemical group at the RNA termini allows distinct RNA subgroups to be distinguished. For instance, treatment of total RNA with terminator exonuclease (TEX), which degrades only processed transcripts (harboring a 5′ P), allows the cDNA library to be enriched for primary transcripts (harboring a 5′ PPP). After TEX treatment, Tobacco Acid Pyrophosphatase (TAP) is used to convert the 5′ PPP to 5′ P, and thus generate RNA 5′ ends suitable for adapter ligation. To enrich the cDNA library with processed transcripts (5′ P RNAs), it can be generated from the untreated RNAs. Comparison of these cDNA libraries by RNA-seq—approach named differential RNA-seq (dRNA-seq)—has been successfully used to map the primary transcriptome of *Helicobacter pylori* (Sharma et al. 2010). In other studies, aimed at identifying processed RNAs, a library containing both primary and processed transcripts has also been included by selectively treating total RNA with a pyrophospohydrolase (e.g. TAP, RppH) (Bischler et al. 2017, Le Rhun et al. 2017, Gill et al. 2018).

Due to the detection limit of most sequencing platforms, cDNA libraries often need to be amplified before sequencing. A limited number of PCR cycles are performed, and several methods are now available to detect and eliminate PCR duplicates during data analysis to avoid bias in transcript abundance determination. For instance, the insertion of unique sequence tags consisting of either defined sequences or random nucleotides (Kivioja et al. 2011, Shiroguchi et al. 2012, Westermann et al. 2012, Fu et al. 2014, 2018) can be used to distinguish between different PCR products.

The amplified cDNAs are then high-throughput sequenced from one extremity (single-end sequencing) or both extremities (pair-end sequencing). The read length *i.e*. number of bases read at once can vary (usually from 50 bp to a maximum of 300 bp for Illumina platforms). A longer read length (more than 10 Kb)—although more error prone—enables transcripts to be mapped more precisely and makes it easier to identify isoforms (Amarasinghe et al. 2020). Another important parameter is the sequencing depth, *i.e*. the number of sequenced reads; by increasing the sequencing depth, it is possible to obtain a more detailed picture of the transcriptome under investigation, for example facilitating the study of weakly expressed transcripts. Overall, the choice of sequencing depth depends on the biological question to be answered.

Prior to mapping the reads (i.e. sequenced cDNAs) to the genome of reference, quality control and normalization steps are required to ensure the accuracy of the results and the comparability of the samples, respectively. The low-quality reads are discarded, as are adapter sequences and duplicated reads due to PCR amplification. The coverage, i.e. the number of reads that are mapped to a defined region of the reference genome, is then calculated and visualized using a genome browser (e.g. IGV and Artemis). Comparison of the expression levels among different samples requires a normalization step, to eliminate biases due to the different gene lengths and sequencing depth. For this purpose, different gene expression measurements such as RPKM (reads per kilobase of transcript per million), FPKM (fragments per kilobase of transcript per million), or TPM (transcript per million reads) are used to account for factors that could affect the comparison of samples. In addition, the number of reads starting (5′ end coverage) or stopping (3′ end coverage) can be calculated at each single nucleotide. This analysis allows the precise mapping of transcript termini, which is highly informative for deciphering the origin of different transcript isoforms, for example derived from RNase activity.

RNA-seq is often used to determine gene expression levels by counting the mapped reads and comparing them among different samples. This allows the identification of genes that are differentially expressed in response to changes in environmental conditions or variations in the expression of a gene under study. RPKM and FPKM are suitable for within-sample comparisons, but not for differential gene expression analysis. Therefore, other normalizing approaches are used for gene count comparisons between samples, such as the approaches included in the DESeq2 (Anders and Huber 2010) and EdgeR (Robinson et al. 2010) Bioconductor packages.

Finally, when designing an RNA-seq experiment, it is crucial to determine the number of replicates. The data reproducibility is affected by technical and biological variability. Technical variability includes for example the RNA extraction, library preparation procedures and to a lesser extent, the sequencing step, which is usually highly reproducible (Mortazavi et al. 2008). Biological variability, which is specific to each experimental set up, is more difficult to control as it cannot be reduced by improving the methodology and the technical procedure. Reproducibility among biological replicates is often assessed using principal component analysis (PCA) (Lever et al. 2017). In PCA, biological replicates subjected to the same experimental conditions are clustered together. To perform a proper statistical analysis, a minimum of three biological replicates is required.

### Transcriptomic approaches to study RNases

The first transcriptomic studies to investigate bacterial RNases were carried out using microarrays (Supplementary Data 1), consisting of gene-specific probes immobilized on a chip capable of binding fluorescently labelled cDNAs derived from the retrotranscription of RNA molecules. The relative abundance of a given transcript was inferred from the intensity of the fluorescence signal generated when the probe hybridized with the labelled cDNA. The immobilized probes were designed based on known open reading frames (ORFs), limiting the study to only these mRNAs. Successively, tiling arrays overcame this limitation, as overlapping probes covering the whole genome were applied. This approach was used, for instance, to investigate the role of RNase E and RNase III in *E. coli* RNA degradation, providing the first transcriptome analysis of a strain deleted of RNase III (Stead et al. 2011) (Supplementary Data 1). Novel RNase III targets were identified and an unprecedented role of RNase III in RNA processing was uncovered. Subsequently, tiling arrays were also applied to *B. subtilis* depleted of RNase Y, RNase J1 or RNase III (Durand et al. 2012). This study revealed a key role of RNase Y and RNase J1 in RNA degradation and a minor role of RNase III in antisense RNA processing.

Probe-dependent approaches have several disadvantages, including the cross-hybridization of the probes, which results in a high level of background signal and a limited detection range due to signal saturation. Another caveat is the prerequisite for information on the genome sequence and annotation. RNA-seq, which provides the sequence of a cDNA molecule as the end result, is a digital quantification approach with a much higher range of detection than probe-based methods. Nowadays, RNA-seq is the method of choice for genome-wide transcript quantification and has been widely used to study bacterial RNases (Supplementary Data 1).

RNA-seq has enormously improved the study of bacterial transcriptome by facilitating the annotation of operons (Creecy and Conway 2015, Yan et al. 2018, Tjaden 2020, Krishnakumar and Ruffing 2022), untranslated regions (Croucher and Thomson 2010) and asRNAs (Toledo-Arana et al. 2009, Sharma et al. 2010, Lasa et al. 2011, Thomason et al. 2015). For instance, transcriptomic analysis of RNA fractions containing either long RNA molecules (>50 nt) or short RNAs (<50 nt) revealed ubiquitous overlapping transcription in *Staphylococcus aureus* (Lasa et al. 2011). At the sites of overlapping transcription, an accumulation of 20 nt-long RNA fragments was observed in the WT strain, but not in an RNase III deletion mutant (∆*rnc*), indicating that these RNA fragments originated from RNase III processing of overlapping transcripts (Lasa et al. 2011).

In addition to calculating steady-state RNA levels, microarray and RNA-seq analyses have been largely used to estimate the RNA half-life (Supplementary Data 1). To this end, the RNA abundance is measured at different time points after RNA synthesis has been stopped in the cells. Transcription arrest is usually achieved by treating the bacteria with rifampicin, i.e. an antibiotic that binds the RNA polymerase and blocks elongation. This strategy has revealed that bacterial RNAs are rapidly degraded, with an average half-life of ∼3 minutes in both *E. coli* (Esquerré et al. 2014, Chen et al. 2015, Dressaire et al. 2018) and *B. subtilis* (Hambraeus et al. 2003). Rifampicin treatment in the WT strain and in a RNase Y deletion strain (∆*rny*) followed by RNA-seq was used to determine whether variations in transcript abundance were due to changes in RNA stability (Khemici et al. 2015). Several rRNAs and transcripts encoding major virulence regulators showed both increased abundance and RNA stability in the ∆*rny* strain, suggesting a possible direct role of RNase Y in the regulation of these targets (Khemici et al. 2015). In a recent preprint, data from RNA-seq at different time points after rifampicin treatment (RIF-seq) were corrected using a hierarchical Bayesian model revealing that RNA half-life in *Salmonella enterica* is shorter than previously reported, with a median RNA half-life of <1 minute at early stationary phase of growth (Jenniches et al. 2023). RBPs, such as such as ProQ and CspC/E, contribute in determining RNA half-life, by promoting RNA stability (Jenniches et al. 2023).

Although transcriptomic studies aimed at identifying the RNA steady-state levels or half-lives are powerful approaches to determine the global role of an RNase in gene expression, these strategies fail to distinguish between direct or indirect RNase targets. Indeed, comparing RNA abundance in the presence and absence of an RNase not only identifies targets that are cleaved and degraded by the RNase under study, but also all RNAs whose steady-state levels vary due to indirect effects, e.g. changes in the transcription levels. For instance, widespread indirect regulation of gene expression has been observed in *E. coli* after deletion of RNase III or RNase E (Stead et al. 2011). In both *S. aureus* and *Streptococcus pyogenes*, RNase Y has been shown to affect indirectly the promoter activity of genes differentially expressed in the ∆*rny* strain compared with the WT strain (Marincola et al. 2012, Broglia et al. 2018). RNases can also influence the stability of an RNA indirectly (i.e. without cleaving), for instance by controlling the levels of sRNAs or helicases, which are post-transcriptional regulators.

Thus, to determine precisely which RNAs are cleaved by an RNase, comparing the abundance of the RNAs is not sufficient.

### Identification of direct RNase targets

A large number of RNA-seq based methods have been developed to identify the direct targets of RNAses and can be classified into two categories: identification of the RNAs bound to an RNase of interest (Table 1), and mapping of RNase cleavage sites (Table 2). In general, RNase-bound targets are identified by purification of the protein of interest, followed by sequencing of the associated RNAs. Depending on the protocol used to retrieve the RNase-RNA complexes, these approaches not only identify RNase targets, but can also provide information on the localization of the interaction, i.e. on binding motifs. On the other hand, mapping the RNA ends generated during a processing event can determine the exact nucleotide position at which an RNase cleaves the target RNAs.

**Table 1.** Transcriptomic studies for the identification of RNase-bound targets

Identification of targets bound to an RNase					
Method^a^	RNase	Mechanism of action^b^	Organisms	Selected main findings	References
Co-IP & RNA-seq	RNase III	dsRNA endoRNase	*S. aureus*	RNase III positively regulates protein synthesis and processes overlapping 5′ UTRs	(Lioliou et al. 2012)
dsRNA immunoprecipitation using J2 antibody			*E. coli*	RNase III plays a key role in the metabolism of dsRNAs *in vivo*	(Lybecker et al. 2014)
RIP-seq			*Streptomyces coelicolor*	RNase III regulon includes 777 transcripts	(Gatewood et al. 2012)
CLASH	RNase E	ssRNA endoRNase	*E. coli*	CLASH identifies sRNA–mRNA duplexes in association with RNase E; the mRNA targets of the Ers41 sRNA were identified	(Waters et al. 2017)
	RNase III	dsRNA endoRNase	*S. aureus*	RNase III stabilizes the pairing between RsaI and RseE sRNAs; RsaI acts as a sponge preventing RseE activity	(McKellar et al. 2022)
CRAC	YbeY	ssRNA endoRNase	*E. coli*	The 3′ UTR of vigR mRNA acts as a *trans*-acting regulatory element by base pairing with different mRNAs and promoting their expression	(Mediati et al. 2022)
				YbeY binds 16S rRNA in vivo; binding sites with sRNAs and mRNAs were not recovered	(McAteer et al. 2018)
RIP-seq		ssRNA (and dsRNA) endoRNase	*Sinorhizobium meliloti*	271 RNAs (more mRNAs than sRNAs) are associated to YbeY	(Saramago et al. 2017)
PNPase Co-IP & RNA-seq	PNPase	3′-to-5′ exoRNase	*E. coli*	PNPase binds and protects sRNAs from degradation	(Bandyra et al. 2016)
GRAD-seq & CLIP-seq	YhaM	3′-to-5′ exoRNase	*Streptococcus pneumoniae*	YhaM interacts and stabilizes sRNAs	(Hör et al. 2020)

a Co-IP, co-immunoprecipitation; RNA-seq, RNA sequencing; CLASH, Cross-linking, Ligation, And Sequencing of Hybrids; CRAC, Cross-Linking and Analysis of cDNAs; RIP-seq, RNA immunoprecipitation and sequencing; GRAD-seq, GRADient profiling by sequencing; CLIP-seq, Cross-Linking ImmunoPrecipitation-high-throughput sequencing.

b dsRNA endoRNase, double-stranded specific endoribonuclease; ssRNA endoRNase, single-stranded specific endoribonuclease; 3′-to-5′ exoRNase, 3′-to-5′ exoribonuclease.

**Table 2.** Methods for the identification of direct RNase targets

Identification of direct RNase targets					
Method^a^	RNase	Mechanism of action^b^	Organisms	Selected main findings	References
RNA-seq coverage comparison	RNase III	dsRNA endoRNase	*E. coli*	Novel RNase III targets coding for metabolic enzymes	(Gordon et al. 2017)
EMOTE	RNase Y	ssRNA endoRNase	*S. aureus*	RNase Y cleaves 99 RNAs with a preference for a guanosine before the processing site in adenosine/uracil rich regions	(Khemici et al. 2015)
	RNases J1/J2	5′-to-3′ exoRNase (RNase J2 also endoRNase)	*S. aureus*	RNase J1 is the main enzyme responsible for the 5′-exonucleolytic degradation and RNase J2 plays a minor role	(Linder et al. 2014)
PARE (mapping of RNA 5′ ends)	RNase III	dsRNA endoRNase	*B. subtilis*	RNase III is involved in the RNA 3′ end maturation of the 16S rRNA	(DiChiara et al. 2016)
Mapping of RNA 5′ ends	RNase III	dsRNA endoRNase	*E. coli*	RNase III cleaves stem favoring G-C or C-G pairing between the two processing sites	(Altuvia et al. 2018)
Mapping of RNA 5′ ends	RNase E	ssRNA endoRNase	*E. coli*	RNase E can initiate RNA degradation though the direct entry pathway	(Clarke et al. 2014)
Mapping of RNA 5′ ends	RNase J	5′-to-3′ exoRNase	*Mycobacterium tuberculosis*	RNase J degrades structured RNA fragments with high GC content	(Martini et al. 2022)
TIER-seq	RNase E	ssRNA endoRNase	*S. enterica*	RNase E displays a 2 nt uridine ruler and cut mechanism	(Chao et al. 2017)
			*Rhodobacter sphaeroides*	RNase E is involved in the oxidative stress response	(Förstner et al. 2018)
			*Vibrio cholera*	RNase E cleaves 3′ UTRs generating sRNAs that act as autoregulatory elements	(Hoyos et al. 2020)
			*Synechocystis sp*.	RNase E displays an adenine at the − 3 and a uridine at the + 2 cleavage signature	(Hoffmann et al. 2021)
REND-seq	RNase Y	ssRNA endoRNase	*B. subtilis*	The Y-complex is necessary for the majority of the RNase Y maturation events	(DeLoughery et al. 2018)
5′ and 3′ end-seq				RNase Y cleaves before an adenosine and after a guanosine in a GC-low sequences	(Taggart et al. 2023)
MORE RNA-seq	MazF	ssRNA endoRNas	*M. tuberculosis*	MazF cleaves the 16S and 23S rRNA and has a UCCUU recognition sequence	(Schifano et al. 2014)
Mapping of RNA 5′ OH ends			*E. coli*	MaxF cleaves most of the mRNAs and r-protein transcripts inhibiting translation	(Culviner and Laub 2018)
Mapping of RNA 5′ ends	RppH	pyrophosphohydrolase	*E. coli*	RppH has a limited number of targets	(Bischler et al. 2017)
Mapping of RNA 3′ and 5′ ends	RNase E	ssRNA endoRNase	*E. coli*	RNase E-generated structured 5′ ends stabilize downstream operon regions	(Dar and Sorek 2018)
Mapping of RNA 3′ ends	PNPase	3′-to-5′ exoRNase	*R. sphaeroides*	5.9% and 9.7% RNA 3′ ends produced by RNase and RNase III, respectively are subsequently degraded by PNPase	(Spanka et al. 2021)
	RNase III	dsRNA endoRNase			
	RNase E	ssRNA endoRNase			
ISCP (mapping of RNA 5′ and 3′ ends)	RNase III	dsRNA endoRNase	*S. pyogenes*	RNase III preferentially cleaves in UTRs and displays a broad nicking activity in vivo	(Le Rhun et al. 2017)
	RNase Y	ssRNA endoRNase		RNase Y generated 320 RNA ends with a preference for a guanosine upstream of the processing site	(Broglia et al. 2020)
	PNPase	3′-to-5′ exoRNase		PNPase is the major 3′-to-5′ exoRNase eliminating RNA fragments	(Lécrivain et al. 2018)
	YhaM	3′-to-5′ exoRNase		YhaM trims on average 3 nt from the majority of the transcripts	(Lécrivain et al. 2018)
	RNase R	3′-to-5′ exoRNase		RNase R has a restricted activity under standard growth condition	(Lécrivain et al. 2018)
3′ end-seq	PNPase	3′-to-5′ exoRNase	*E. coli*	The deletion of PNPase leads to an increase in internal RNA 3′ ends corresponding to RNA decay fragments produced by RNase E	(Herzel et al. 2022)

a PARE, Parallel Analysis of RNA Ends; ISCP, Identification of Specific Cleavage Positions; TIER-seq, Transiently Inactivating an Endoribonuclease followed by RNA sequencing; EMOTE, Exact Mapping of TranscriptOme Ends; REND-seq, RNA end-enrichment sequencing; MORE RNA-seq, Mapping by OveRExpression RNA sequencing.

b dsRNA endoRNase, double-stranded specific endoribonuclease; ssRNA endoRNase, single-stranded specific endoribonuclease; 3′-to-5′ exoRNase, 3′-to-5′ exoribonuclease.

#### RNase pull-down and sequencing of the associated RNAs

Co-IP of a tagged RNase and associated RNAs, followed by RNA-seq, has been applied to many RNases, in order to reveal the direct targets bound to the RNase under study (Table 1). Several variations in the procedure have been introduced, resulting in multiple strategies with or without a cross-linking step. The protein-RNA cross-linking, generally performed by ultraviolet (UV) irradiation or formaldehyde treatment, promotes the formation of covalent bonds between a protein and the bound RNAs. This makes it possible to identify the interaction site between the two molecules after trimming unbound RNA (Saliba, C Santos, and Vogel 2017).

**Figure 2.**
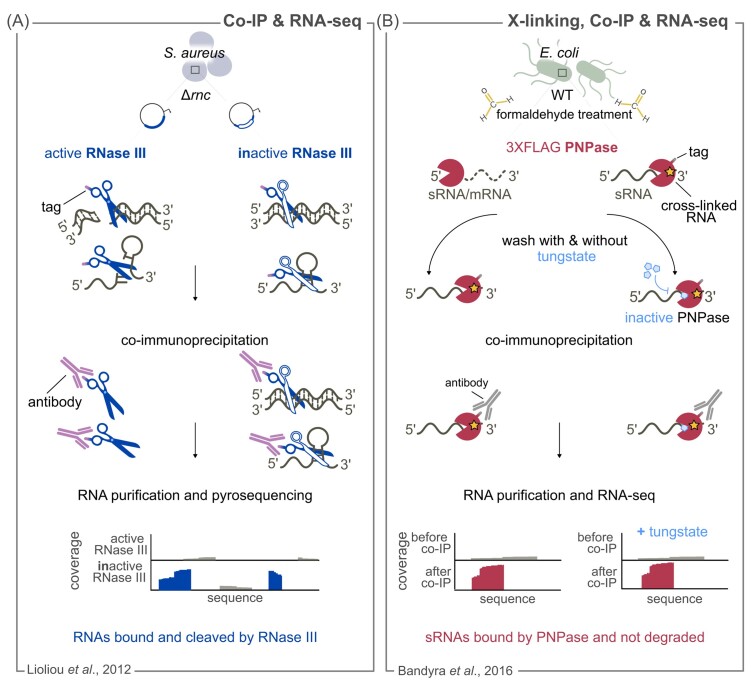
Co-IP of RNAs bound to an RNase followed by RNA-seq to uncover the direct RNase targets. **(A)** and **(B)** Schematic representation of methods based on Co-IP of a tagged RNase and associated RNAs, followed by sequencing of the associated RNAs. A schematic representation of the coverage profiles obtained by RNA-seq is shown for each approach. **(A)** The *S. aureus rnc* gene encoding RNase III is deleted (∆*rnc*) and epitope-tagged active RNase III (blue scissors) or catalytically inactive RNase III (blue and white) are expressed from a plasmid under control of a Cd^2+^ inducible promoter. The inactive RNase III variant is able to bind the target RNA but not to cleave it. The RNAs bound to RNase III are co-immunoprecipitated with an antibody recognizing the epitope and used for library preparation and subsequent pyrosequencing. The reads corresponding to RNAs from the Co-IP with the inactive RNase III, but not with the active RNase III, are shown in blue. **(B)***In vivo* formaldehyde treatment of *E. coli* cells covalently cross-links RNA to proteins; the yellow star depicts the covalent bond between the two molecules. PNPase can degrade RNA molecules (dashed line) or bind to sRNAs, protecting them from degradation. Co-purification of a 3xFLAG PNPase variant is carried out in the presence or absence of tungstate (blue pentagon). This metal is thought to bind to the catalytic center of PNPase, specifically to the phosphate interacting site, blocking the PNPase catalytic activity. Co-immunoprecipitated RNAs are used for library preparation and RNA-seq. A schematic representation of the coverage profiles obtained by RNA-seq is shown for each method. sRNAs were found to be the preferred species stably associated with PNPase.

Co-IP of an active and catalytically inactive version of the RNase III enzyme combined with RNA-seq of the bound RNAs was used to identify the first global mapping of direct RNase III targets in *S. aureus* (Lioliou et al. 2012) (Fig. 2A) and *S. coelicolor* (Gatewood et al. 2012). This approach was initially developed to profile genome-wide sRNAs and possible target mRNAs bound to Hfq (Sittka et al. 2008, 2009). Lioliou et al. exploit the Co-IP of histidine epitope-tagged WT RNase III and a catalytic inactive variant, yet capable of binding its substrates (Fig. 2A). The target RNAs bound to the RNase III mutant unable to cleave are captured, converted to cDNAs and sequenced. In contrast, in the presence of an active RNase III, the fragments generated during processing are displaced and eventually degraded (Fig. 2A). As a control, an untagged version of active RNase III was included in the analysis, to monitor the RNAs that are nonspecifically bound during the co-IP procedure (Lioliou et al. 2012).

In addition to the canonical role of RNase III in rRNA maturation, this work uncovered several novel RNase III targets. For instance, RNase III has been shown to positively regulate the synthesis of the major cold shock protein CspA by processing the 5′ untranslated regions (5′ UTR) of *cspA* mRNA and producing a shorter stabilized mRNA isoform that is more efficiently translated. This work also revealed that several different asRNAs, corresponding to 44% of annotated genes, were bound to inactive RNase III variants, confirming the role of this endoRNase in antisense RNA-mediated regulation.

Although this method gives a global overview of RNase III-bound and RNase III-cleaved RNAs, it provides only limited information on the position of RNase III processing events. Indeed, the position of RNase III processing sites can only be deduced if one of the cleaved products remains bound to the active variant of RNase III (WT RNase III). For instance, in the *rnc* mRNA (coding for RNase III), a 5′ end of the product detected by sequencing after co-IP coincided with the RNase III processing site validated *in vitro*. Notably, RNase III has the ability to function in a noncatalytic mode and can bind some target RNAs without cleaving them (Calin-Jageman and Nicholson 2003). Consequently, the RNAs bound to the inactive variants are not necessarily cleaved by RNase III, and *in vitro* validation of the processing events is required. RNase Co-IP followed by RNA-seq was also successfully applied to the 3′-to-5′ exoRNase PNPase in *E. coli* (Bandyra et al. 2016) (Fig. 2B). Flagged PNPase was cross-linked to its targets by *in vivo* treatment with formaldehyde. This cross-linker, which rapidly penetrates cells, stabilizes macromolecular complexes (including protein-RNA complexes) by forming reversible covalent bonds (Sutherland et al. 2008, Ramanathan et al. 2019). RNA-seq analysis performed before and after PNPase Co-IP revealed that sRNAs are the preferred RNA species stably associated with PNPase (Fig. 2B). sRNAs were specifically associated with PNPase, as Northern blot analysis revealed no sRNA enrichment after Co-IP of an untagged PNPase variant. To determine whether these sRNAs were substrates of the PNPase exoribonucleolytic activity, cells harvested after cross-linking were treated with an inhibitor of PNPase catalytic activity (*i.e*. tungstate) (Symmons et al. 2000) (Fig. 2B). RNA-seq analysis before and after PNPase Co-IP revealed that the impaired degradative activity does not alter the amount of sRNAs associated with PNPase, suggesting that the regulatory RNAs are bound, but not degraded by this exoRNase. This study highlights the role of PNPase as an RBP that protects sRNAs from degradation (Bandyra et al. 2016).

The principle of specifically pulling down the protein of interest and sequencing the associated RNAs has been widely applied to the study of RBPs (Saliba, C Santos, and Vogel 2017). Cross-linking and analysis of cDNAs (CRAC) (Tree et al. 2014), RNA interaction by ligation and sequencing (RIL-seq) (Melamed et al. 2019), cross-linking immunoprecipitation-high-throughput sequencing (CLIP-seq) (Holmqvist et al. 2016, 2018) were first successfully used to identify RNAs bound to Hfq, CsrA, and ProQ (Hör, Gorski, and Vogel 2018). Similar approaches have also been applied to RNases for mapping their direct targets. All these methods include a step of *in vivo* UV protein-RNA cross-linking prior to co-purification. Compared with formaldehyde, UV light is considered a more specific cross-linker, first because it favors formation of a covalent bound only when there is no distance between the interacting molecules, and secondly because it does not promote cross-linking between interacting proteins (Ramanathan et al. 2019). However, the efficiency of UV cross-linking can be affected by the amino acid and RNA sequence, e.g. uridine sequences are preferred (Sugimoto et al. 2012). Methods based on UV cross-linking allow more stringent denaturation conditions during the purification steps and also trim the protein bound-RNAs, giving the opportunity to fine-map the RNA binding sites.

**Figure 3.**
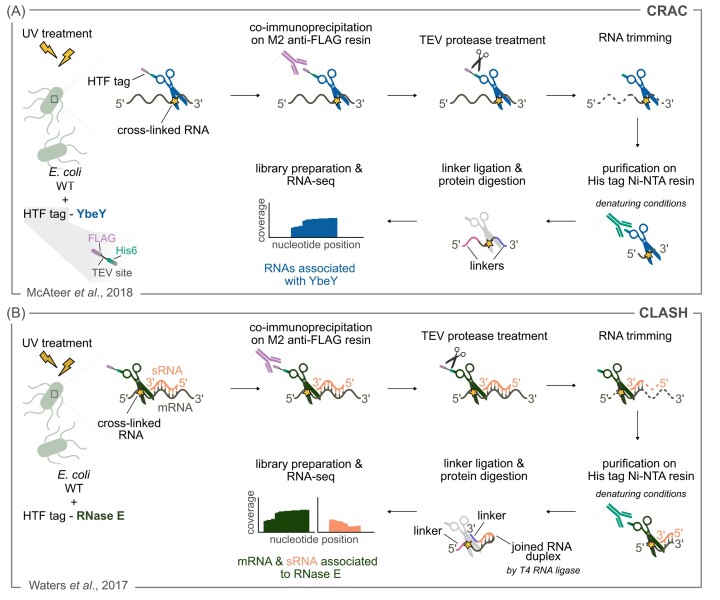
UV irradiation prior to RNase co-immunoprecipitation and RNA-seq to identify RNase targets. **(A)** and **(B)***E. coli* WT cells are UV-irradiated to promote the cross-linking (depicted by the yellow star) of RNA molecules with the interacting protein. The RNase of interest is co-immunoprecipitated using a dual affinity tag composed of a FLAG tag, a TEV protease site and a His6 tag (HTF tag) (shown in A). The RNase is first purified using the FLAG tag, which is then removed by the TEV protease. A mix of RNases is used to trim the cross-linked RNAs (dotted lines), which are subsequently co-purified using the His6 tag, under denaturing conditions. Linkers are ligated to the extremities of the cross-linked RNAs, and after complex purification and protein digestion steps, the resulting RNAs are used for library preparation and RNA-seq analysis. **(A)** CRAC: UV cross-linking and analysis of cDNAs. This method is used to identify the RNAs bound to YbeY. **(B)** CLASH. This technique is used to identify RNase E-associated RNA duplexes. The interacting RNAs (e.g. a sRNA and mRNA) associated with RNase E are ligated into a single hybrid molecule, using T4 RNA ligase.

CRAC analysis in enterohemorrhagic *E. coli* has been applied to identify the RNA binding sites of YbeY, an endoRNase involved in ribosome maturation (McAteer et al. 2018). To ensure that only RNAs covalently linked to the protein of interest are purified, the protein of interest is labeled with a bipartite tag and the purification is done in highly stringent purification conditions (Fig. 3A). The dual affinity tag, consisting of a TEV protease site surrounded by His6 and FLAG tags (HTF dual tag), was fused to the C-terminus of YbeY (Fig. 3A) (Tree et al. 2014). A cDNA library for RNA-seq was generated from the eluted RNAs. A strain expressing an untagged YbeY variant was included in the experiment as a control. A total of nine YbeY RNA-binding sites were identified, including eight located in rRNAs and one in the tRNA^leu^. The regions of contacts between YbeY and the 16S rRNA support the already known role of YbeY in the 16S rRNA 3′ end maturation (Jacob et al. 2013). The authors observed that the effect of YbeY on the expression of the type III secretion system was not due to a direct activity of YbeY, but rather resulted from reduced levels of mature ribosomes. In a previous study, Co-IP of a flag-tagged variant of YbeY from *S. meliloti* followed by RNA-seq, identified over 200 RNAs associated with this RNase (Saramago et al. 2017). The targets identified included mRNAs and, to a lesser extent, asRNAs. YbeY was initially proposed to be involved in sRNA-mediated regulation of gene expression, possibly acting as an sRNA chaperone, similarly to Hfq (Rasouly et al. 2010, Pandey et al. 2011, 2014; Leskinen et al. 2015). However, these two studies, aimed at identifying the RNAs bound to YbeY, revealed that this endoRNase probably does not act as an sRNA chaperone.

CLASH was first established to identify RNA hybrids bound to RNase E in *E. coli* (Waters et al. 2017). Enterobacteriaceae sRNAs typically base-pair with their target mRNAs via the Hfq chaperone; the complex formed is then able to recruit RNase E, which initiates the degradation of the target mRNAs (Gottesman and Storz 2011, Bandyra et al. 2012). The rationale behind this study relies on the hypothesis that the sRNA-mRNA complex is transiently associated with RNase E. As in the CRAC analysis, an HTF-tagged version of RNase E was expressed from the chromosome of enterohaemorrhagic *E. coli. In vivo* UV cross-linking ensured that the sRNA-mRNA duplexes remained covalently bound to RNase E prior to co-purification (Fig. 3B). The RNase E-bound RNAs were trimmed by RNases and ligated to RNA linkers for Illumina sequencing by T4 RNA ligase treatment. If the RNAs bound to RNase E participate in the interaction with sRNAs, the ligase treatment results in hybrid RNA molecules composed of a sRNA and the corresponding target mRNA. After purification of these complexes, RNase E is digested by proteases and the co-purified RNAs are used for library preparation and RNA-seq (Fig. 3B). This study shed light on the mechanism of sRNA-mediated degradation of target mRNAs by RNase E and identified several tRNA-associated sRNAs that function as sRNA sponges. Overall, the sequenced reads mapped to 75% of the annotated genes, consistent with a major role of RNase E in RNA degradation. Peaks of reads mapped in the CLASH experiment were found in proximity to known RNase E cleavage sites (Clarke et al. 2014). Poly(A) tails were enriched at some known RNase E cleavage sites, indicating that the RNA 3′ end of the RNase E-bound fragment derived from a RNase E processing event *in vivo*, followed by the addition of an oligonucleotide tail. However, in the majority of cases, the RNA ends of the RNase-E bound fragments did not correspond to the positions at which RNase E cleaved the target. This may be explained by the fact that, during the library preparation, the RNAs are trimmed by other RNases.

Recently, the CLASH approach was used to reveal RNase III-associated RNA duplexes in a methicillin-resistant *S. aureus* (MRSA), providing insight into the role of RNA-mediated regulation in the development of antibiotic resistance and during infection (McKellar et al. 2022, Mediati et al. 2022). The CLASH protocol was carried out by comparing a strain expressing RNase III tagged with the HTF dual tag and a strain expressing an untagged RNase III. CLASH revealed that RNase III is strongly associated with long and structured UTRs and highlighted several functional RNA-RNA interactions, identifying novel target sRNAs. CLASH was combined with mapping of both 5′ and 3′ ends using dRNA-seq and Term-seq, respectively (Mediati et al. 2022). Coupling mapping of the transcriptome architecture with CLASH not only identified novel regulatory RNAs, but also deciphered their mechanism of action. RNase III-CLASH in MRSA has uncovered a novel mechanism of intermediate vancomycin resistance based on the *trans*-acting 3′ UTR of *vigR* mRNA.

In a second study, RNase III CLASH was performed in MRSA using growth media mimicking different host environments (McKellar et al. 2022). This study revealed novel environment-specific sRNA-RNA interactions, indicating that RNase III plays a much more important role than expected during infection. Interestingly, the authors reported that RNase III does not process the duplex formed by the RsaI and RseE sRNAs, but rather functions as a chaperone that stabilizes the interaction between them. RNase III CLASH also revealed that the expression of several toxins is controlled by sRNAs, which often base pair close to the Shine-Dalgarno sequence and probably induce RNase III processing of the target mRNA.

These two studies show how the coupling of methods for RNase target identification with standard RNA-seq under infection conditions or on clinical isolates can uncover new mechanisms of virulence regulation.

#### Gradient profiling by sequencing

**Figure 4.**
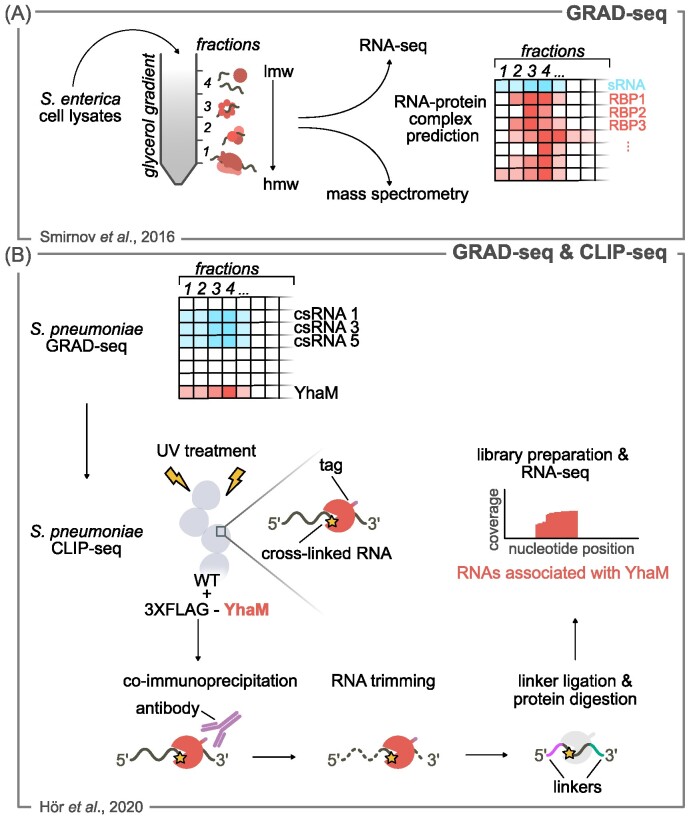
GRAD-seq coupled with CLIP-seq to identify RNase-associated RNAs. **(A)** GRAD-seq: gradient fractionation followed by RNA-seq. *S. enterica* cell lysate is fractionated according to the molecular weight (lmw, low molecular weight; hmw, high molecular weight) using a glycerol gradient. Each fraction (indicated with numbers) is analysed by RNA-seq and mass spectrometry. RNA-protein complexes are predicted on the basis of the RNAs and proteins found in the same fraction. **(B)** Grad-seq in *S. pneumoniae* revealed that Cia-dependent sRNAs (csRNAs) are strongly associated with YhaM. CLIP-seq: cross-linking immunoprecipitation-high-throughput sequencing was used to confirm the association of csRNAs with YhaM. WT *S. pneumoniae* cells are UV-irradiated to promote cross-linking (yellow star) of RNAs with YhaM fused to a FLAG tag. YhaM is co-immunoprecipitated and the bound RNAs are trimmed outside of the binding region. Linkers are added to the trimmed RNAs and the cross-linked protein is digested by proteases. The resulting RNAs are used for library preparation and RNA-seq analysis. A schematic representation of the coverage profile obtained by RNA-seq is shown.

Gradient profiling by sequencing (GRAD-seq) has recently been used to discover RNA-protein complexes by fractionating bacterial lysates on linear glycerol gradient and analyzing each fraction by RNA-seq and mass spectrometry (Smirnov et al. 2016) (Fig. 4A). RNAs interacting with a particular protein are clustered within the same fraction, which is then examined by mass spectrometry to identify the shared interaction partner (Fig. 4A). Unlike pull-down approaches, which are applied to a specific RBP or RNase of interest, GRAD-seq enables the identification of RNA-protein complexes without a prior knowledge of the protein. This strategy therefore opens the possibility of identifying new proteins involved in RNA binding and/or processing. Grad-seq has unveiled invaluable new information on RNA-binding proteins in enteric bacteria, enabling the identification of ProQ as a novel sRNA chaperone in addition to Hfq (Smirnov et al. 2016, Attaiech et al. 2017).

This approach was recently applied to *S. pneumoniae* to identify novel RBPs (Hör et al. 2020) (Fig. 4B). Different sRNA clusters were defined on the basis of their migration pattern and one of them included the five csRNAs, which are involved in the regulation of pneumococcal competence (Halfmann et al. 2007). The csRNA-interacting proteins were pulled down, using *in vitro* synthesized sRNAs harboring a tag, and analysed by mass spectrometry. Among the interacting proteins, several RNases (e.g. RNases J1 and J2) were identified and in particular YhaM (also known as Cbf1), which functions as a 3′-to-5′ exoRNase, was the most enriched protein. The YhaM-csRNA interaction was confirmed by CLIP-seq using an YhaM flag tagged version (Fig. 4B). YhaM was shown to trim only a few nucleotides at the csRNA 3′ ends, as previously shown in *S. pyogenes* for the majority of RNAs (Lécrivain et al. 2018). As YhaM has been shown to exert a protective role in preventing csRNA degradation, the authors concluded that following RNA trimming, the YhaM-csRNA persists as a stable complex stabilizing the csRNAs. This study provides an example of how GRAD-seq followed by downstream approaches (e.g. CLIP-seq) might be a powerful technique not only for studying RBPs, but also RNases.

#### Immunoprecipitation and sequencing of double-stranded RNAs

Whereas the above-described methods require the selection of a protein, which is then Co-IPed with the interacting RNAs, Lybecker et al. have used an alternative approach to identify RNase III targets based on the isolation of a specific class of RNAs.

**Figure 5.**
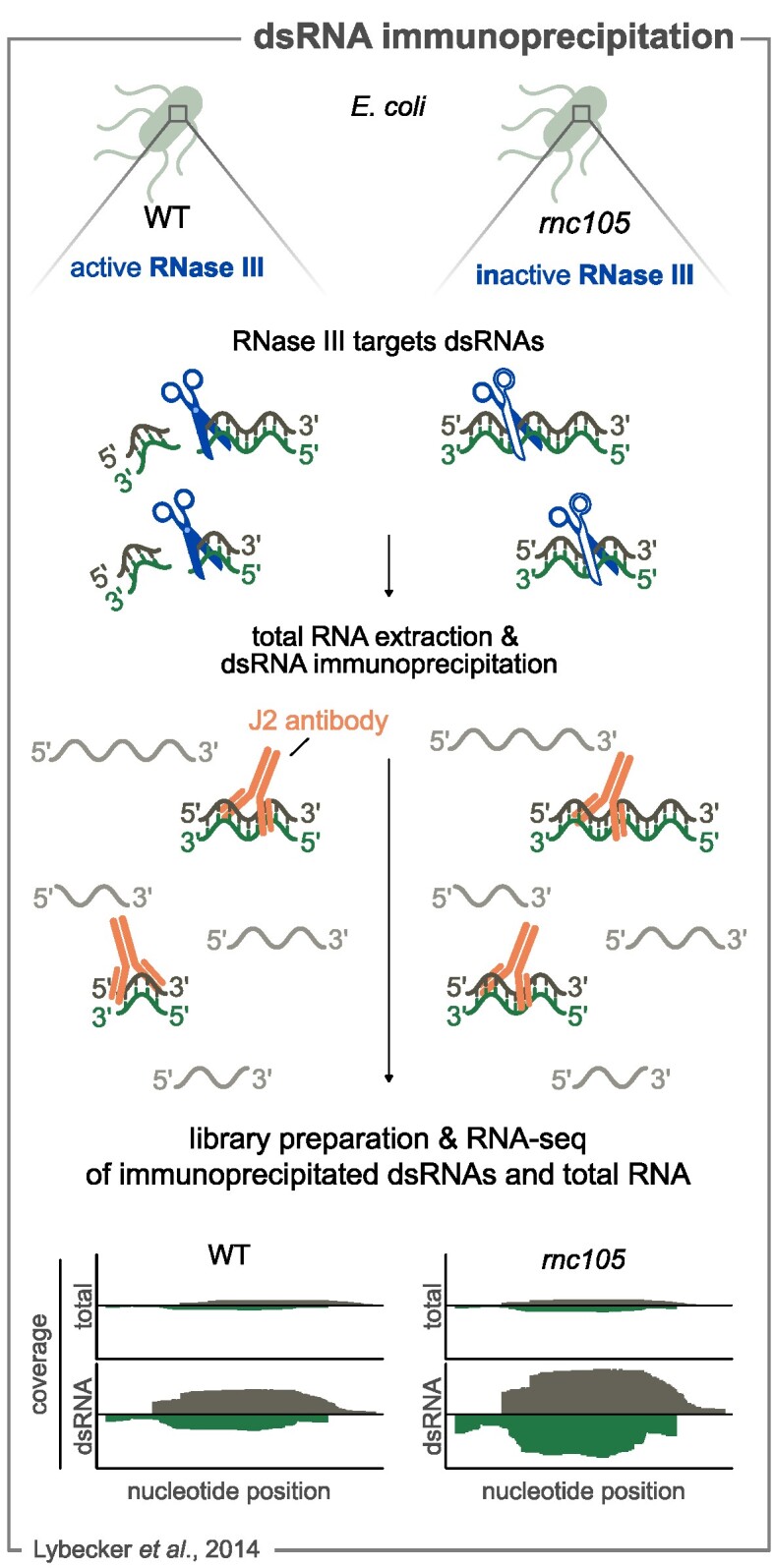
dsRNA immunoprecipitation coupled with RNA-seq analysis to unravel the RNase targetome. *E. coli* WT expresses an active RNase III (blue scissors), capable of cleaving double-strand RNAs (dsRNAs). The *rnc105* strain expresses a catalytically inactive RNase III variant, which binds to target dsRNAs but does not cleave them. Total RNA is extracted from both strains and the dsRNAs are immunoprecipitated using the J2 antibody, which recognizes dsRNAs but not ssRNAs (in gray). The purified dsRNAs and total RNA are used for library preparation and RNA-seq analysis. A schematic drawing of the coverages (gray for positive strand and green for negative strand) is shown. An increase in dsRNA coverage is generally observed after J2 treatment compared with the total RNA sample in WT and *rnc105* strains. The level of dsRNA coverage is generally higher in the *rnc105* strain compared to WT, suggesting that these dsRNAs are targeted by RNase III. A schematic representation of the coverage profiles obtained by RNA-seq is shown.

RNase III specifically cleaves double-stranded RNAs (dsRNAs), consisting of duplexes formed by the paring of sense and antisense RNAs or structured RNAs (e.g. stem-loops). This characteristic was exploited to selectively identify the dsRNAs that are targeted by RNase III in *E. coli* (Fig. 5). The method relies on the specificity of the J2 antibody, which is able to bind dsRNAs of at least 40-nucleotides (nt) long, irrespective of their sequence and nucleotide composition (Lybecker et al. 2014) (Fig. 5). The dsRNAs were immunoprecipitated by incubating the J2 antibody with total RNA extracted from a WT strain and a strain in which RNase III is catalytically inactive (*rnc105*) (Fig. 5).

As a control, total RNA from both strains was also sequenced. In total, over 200 dsRNAs were enriched only in the *rnc105* strain, indicating that RNase III is responsible for the degradation of these dsRNAs in the WT strain. This study confirmed the key role of RNase III in the metabolism of dsRNAs *in vivo*, as previously observed in *S. aureus* (Lasa et al. 2011).

The majority of the immunoprecipitated dsRNAs were detected in the 5′ UTR, suggesting that regulation of gene expression via asRNAs and RNase III processing activity takes place primarly in the transcript 5′ end regions. A significant number of dsRNAs processed by RNase III also derived from antisense transcription of sRNAs.

This method revealed the presence of a large amount of overlapping RNAs that are processed by RNase III. As it relies specifically on the isolation of dsRNAs, it also provides evidence that overlapping sense and antisense RNAs are present simultaneously in a bacterial cell. RNase III has been shown to be involved in processing of overlapping transcripts in *S. aureus*, generating a pool of approximately 20-nt long RNAs (Lasa et al. 2012). This method successfully describes the role of RNase III in dsRNA metabolism; however, it is not suitable to determine the exact positions at which RNase III processes its substrates.

## Genome-wide mapping of RNA ends

RNA-seq not only enables genome-wide quantification of transcripts, but also, thanks to single-nucleotide resolution, accurate mapping of the transcript boundaries. By mapping RNA ends in a genome-wide manner, we can uncover many facets of RNA metabolism, including transcription termination, degradation and regulation. For instance, mapping of RNA ends can facilitate the discovery of novel RNA regulatory elements. High-precision mapping of RNA 3′ ends (TERM-seq) has been developed to identify novel riboswitches and attenuators in pathogenic and commensal bacteria (Dar et al. 2016, Adams et al. 2021). Regulatory functions for RNA fragments, derived from premature termination or mRNA processing, were discovered by global mapping of 3′ ends in *E. coli* (Adams et al. 2021).

The investigation of RNA termination events in bacteria has advanced considerably thanks to the ability to accurately map RNA ends. Sequencing of 5′ and 3′ ends (SEnd-seq) has been used to simultaneously map transcription start and end sites in *E. coli*. (Ju et al. 2019). SEnd-seq revealed ubiquitous overlapping bidirectional transcriptional termination due to convergent transcription. The mapping of RNA ends in *Borrelia burgdorferi* has recently provided valuable insights into transcription termination processes and the identification of potential regulatory elements (Petroni et al. 2023).

Understanding the precise positions of RNA termini plays a pivotal role in the study of RNA degradation pathways in bacteria. Term-seq was adopted by Dar et al., to study Rho-dependent or -independent transcription termination (Dar and Sorek 2018a), and to decipher the mechanism of differential RNA degradation of RNAs originated from the same polycistronic transcript (Dar and Sorek 2018b). RNA-seq profiling of RNA 3′ ends derived either from total RNA or from RNA polymerase-associated RNAs (i.e. nascent RNAs) has provided an atlas of intermediate decay fragments in *E. coli* and *B. subtilis*. Intermediate decay fragments, although more abundant than their parental transcripts, do not contribute to protein production, as they are less associated with ribosomes (Herzel et al. 2022).

### Identification of RNase cleavage sites

**Figure 6.**
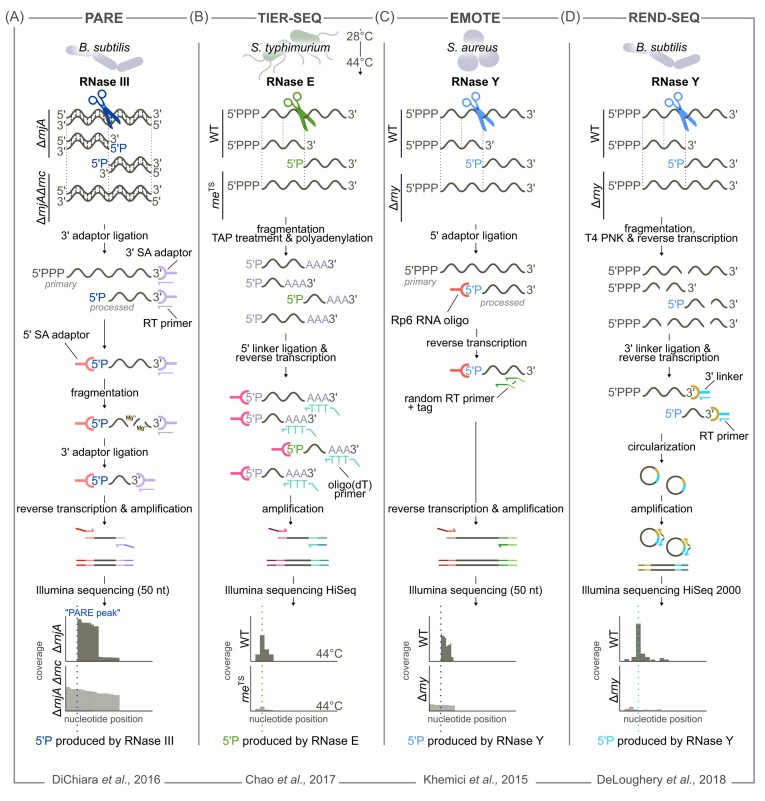
Genome-wide mapping of RNA 5′ ends to identify endoRNase specific cleavage sites. **(A–D)** Schematic representation of selected RNA-seq methods based on mapping o RNA 5′ ends to identify endoRNase processing sites.Only critical steps in the library preparation are shown, while others (e.g. DNase treatment and rRNA removal) are not represented. A graphical representation of the coverage (i.e . the number of reads mapped to specific location) is shown for each method. Processing events are identified by screening for locations showing a decrease in coverage in the strain deleted of the RNase under study compared with the WT strain. **(A)** PARE: Parallel Analysis of RNA ends. Total RNA from *B. subtilis* is extracted from both an RNase J1 deletion mutant (Δ*rnjA*), which is considered here as a reference strain, and the RNase J1 and RNase III double deletion mutant (Δ*rnjA*Δ*rnc*). In a first step, the 3′ SR adapter is ligated to the RNA 3′ ends and the RT primer is hybridized; in a second step, the 5′ SR adapter is also ligated. The RNAs are fragmented using magnesium and the ligation of the 3′ SR adapter is repeated.Size-selected cDNAswere PCR amplified and subsequently used for 50-nt single-end RNA-seq. An RNA 5′ P end is identified as the first nucleotide of a 50 nt-read. **(B)** TIER-SEQ: Transiently inactivating an endoribonuclease followed by RNA-seq. RNA-seq is performed before and after RNase E inactivation using RNA from a WT strain and a temperature-sensitive RNase E mutant ( *rne*^TS^) in *Salmonella typhimurium*. When the temperature is shifted from 28°C to 44°C, RNase E retains its activity in the WT strain but not in the *rne*^TS^ mutant. RNAs are converted from 5′ PPP to 5′ P RNAs using TAP and subsequently polyadenylated; an RNA adapter is then ligated to the RNA 5′ P ends. RNAs are retrotranscribed using an oligo(dT)-adapter primer and amplified. Cleavage positions are identified by comparing the number of reads per nucleotide in the WT and *rne*^TS^ stains at 44°C. The 5′ end of each read was isolated to calculate the number of all 5′ reads mapped per nucleotide. To localize RNase E processing sites, the number of reads per nucleotide of the WT strain grown at a nonpermissive temperature was compared, using DESeq2, to that of the RNase E thermosensitive mutant grown under the same conditions. **(C)** EMOTE. *Staphylococcus aureus*total RNA is extracted from both a WT strain and the *rny* deletion mutant strain (Δ*rny*) and ligated to the Rp6 oligo. This reaction can occur only in the presence of a 5′ P end of the RNA, so only processed transcripts will be ligated with this oligo. The ligated RNAs are reverse transcribed (RT) using a random primer containing a tag at the 5′ end. The cDNA is PCR-amplified with primers harboring adapters for Illumina sequencing . The first base of the mapped sequence corresponds to the location of an RNA 5′ P end. **(D)** REND-SEQ: RNA end-enrichment sequencing. *S. aureus* total RNA is extracted from both a WT strain and the *rny* deletion mutant strain (Δ*rny*), fragmented, size-selected (20–40 nt) and dephosphorylated using T4 polynucleotide kinase. A 3′ linker is ligated to the RNA 3′ ends and the RNA is retrotranscribed with an RT primer. The cDNA produced is circularized and used for amplification. The 5′ and 3′ end read counts were determined and the RNA termini were annotated by identifying single nucleotide positions with an increase in the REND-seq read count (i.e. sharp peaks in the coverage profile).

The identification of RNA ends (5′ and 3′ ends) opens the possibility of specifically detecting RNase processing sites and several tailored RNA-seq approaches have been developed for genome-wide identification of the cleavage sites of an RNase (Table 2). Most of these methods rely solely on mapping RNA 5′ ends (Fig. 6) to infer the location of an RNase processing site without taking into account information on the RNA 3′ ends. The identification of RNA 3′ ends has only recently been applied to the study of bacterial RNases. Overall, recent advances in RNA-seq technologies have broadened the approaches used to identify RNase processing sites, improving our current understanding of RNase targetomes in bacteria and revealing new RNase functions. The following sections present the methods developed for the identification of bacterial RNase cleavage sites.

#### RNase III

RNase III is a dsRNA-specific endoribonuclease that cleaves the phosphodiester bond between two nucleotides, leaving 3′ OH and 5′ P termini and a 3′ overhang of 2 nt. Therefore, ideally, RNase III processing results in four RNA ends comprising two newly generated 5′ P.

Gordon *et al*., sought to identify the putative RNase III processing positions in *E. coli* by sequencing RNAs harvested from a WT strain and a *rnc* (encoding RNase III) deletion mutant strain (∆*rnc*), before and after the addition of rifampicin (Gordon et al. 2017). Comparison of the read coverage between the WT and ∆*rnc* strains revealed the approximate position of the RNase III processing site. Indeed, at known processing positions, a reduced coverage is detected in the WT strain compared to the ∆*rnc* strain, as a result of the degradation of the RNase III-generated fragment. Because of the rationale used, only cleavage sites responsible for the reduction in RNA abundance in the WT strain are mapped and, consequently, processing sites that do not lead to fragment destabilization cannot be identified. This method revealed new RNase III targets encoding metabolic enzymes (e.g. pyruvate dehydrogenase complex) and a role for RNase III in the initiation of decay transcripts derived from premature termination. Although this method allows approximate localization of the RNase III cleavage sites, it does not provide information on the exact position of the cleavage.

The PARE RNA-seq protocol was then developed to specifically map the RNA 5′ P ends generated by RNase III processing in *B. subtilis* (DiChiara et al. 2016) (Fig. 6A). This approach takes advantage of (i) the fact that RNase III produces RNA 5′ P ends and (ii) the possibility of specifically selecting the 5′ P RNAs during library preparation (Fig. 6A). Strains deleted or not of *rnc* were compared. To capture the original RNA 5′ P ends generated by RNase III, the reference and ∆*rnc* strains were deleted of *rnjA*, coding for RNase J1, which could potentially degrade the products derived from RNase III processing. Overall, PARE identified 53 and 5 putative RNase III processing sites in mRNAs and intergenic regions, respectively. Of the processing events in target mRNAs, only a few required the pairing of an antisense RNA, in contrast to previous studies in which RNase III was shown to play a key role in RNA duplex degradation (Lasa et al. 2012, Lioliou et al. 2012). PARE analysis provides evidence that the processing required for the (almost) final 3′ end maturation of the 16S rRNA is due to an endoRNase activity and not to trimming by exoRNase(s), as originally predicted. Finally, PARE also revealed an enrichment of RNA 5′ ends in the terminal region of several ORFs. It was concluded that RNase J1 plays an important role in the degradation of RNA fragments containing a terminator region.

Subsequently, Altuvia and colleagues also took advantage of the ability to capture the 5′ P ends of RNAs generated by RNase III to globally map the targetome of this dsRNA-dependent endoRNase in *E. coli* (Altuvia et al. 2018). Similar to the PARE protocol, the cDNA library was obtained by selecting RNAs longer than 100 nt and ligating distinct adapter sequences to the RNA 3′ and then 5′ ends. Since RNase III often processes duplex RNAs formed by an mRNA and an sRNA, the authors reasoned that the products resulting from this processing would be too short to be detected by RNA-seq. Consequently, two cDNA libraries were generated: in one, long RNAs (>100 nt) were selected and fragmented for library preparation, in the other, short RNAs (<16 nt) were selected and used for adapter ligation and cDNA synthesis without fragmentation.

Since the 5′ P of a fragment corresponds to the first nucleotide of a read, the number of ‘start reads’ mapped in the WT strain and in a strain expressing an inactive version of RNase III were calculated and compared using the Bioconductor DESeq2. A major advantage of this approach over PARE is that it includes statistical power for more robust genome-wide identification of RNase III processing sites. This analysis revealed in total of 1003 RNase III processing sites, which were mainly found in intergenic regions, UTRs and noncoding RNAs.

RNase III consists of a homodimer in which the catalytic site of each subunit can independently cleave one of the RNA strands, generating a double strand break. The distance between the two cleavage sites (i.e. structural distance) defines the length of the nucleotide overhang, which typically consists of 2 nt protruding from the 3′ end. By analyzing the region surrounding the mapped RNase III processing sites, the authors unraveled new features of RNase III's mode of action *in vivo*. RNase III targets were classified according to whether only one or both processing sites were detected. In the case of two cleavage sites per target, the authors calculated the structural distance, which generally consisted of 2 nt, as previously shown. However, a noncanonical RNase III structural distance was observed when the two RNase III cleavage sites did not occur during the same processing event. Stem structures were predicted in the targets harboring two RNase III cleavages sites with a canonical structural distance. The alignment of these structures revealed that RNase III recognizes a specific base-pairing within the stem. Indeed, this RNase cleaves the stem, favoring G-C or C-G pairing between the two processing sites. The authors hypothesized that single processing events could be caused either by the inability to detect the other processing site, or by the nicking activity of RNase III (i.e. cleavage of only one RNA strand) (Court et al. 2013), or by the processing of an intermolecular duplex.

**Figure 7.**
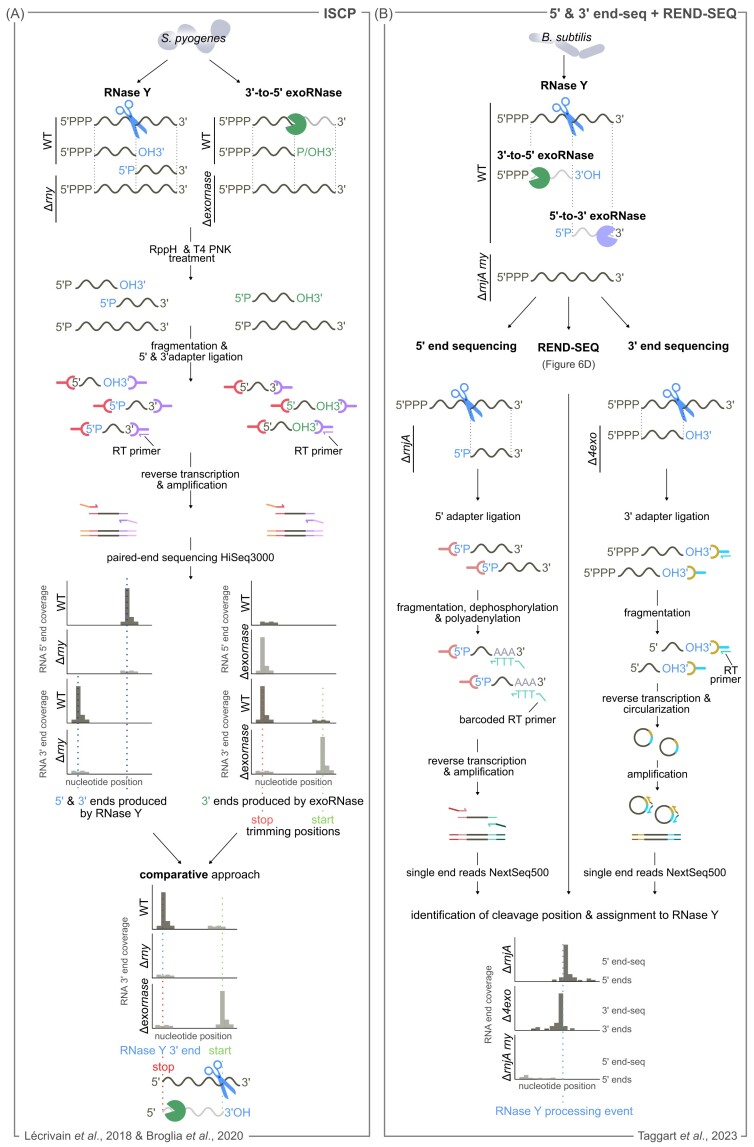
Mapping of the 5′ and 3′ ends of RNA to localize endo and exoRNase processing events. **(A)** ISCP. Total RNA is extracted from a WT strain and a strain deleted of the RNase under study, i.e. RNase Y (Δ*rny*) or a 3′-to-5′ exoRNase (PNPase, YhaM or RNase R), dephosphorylated using T4 polynucleotide kinase (T4 PNK) and treated with a pyrophospohydrolase (RppH), which converts the RNA 5′ PPP ends into RNA 5′ P ends, therefore both processed transcripts and primary transcripts are included in the library. After fragmentation, the RNAs are ligated to both 5′ and 3′ adapters and retrotranscribed. cDNAs are amplified and sequenced using pair-end illumina sequencing. Processing events are identified by screening for locations showing a decrease in coverage in the strain deleted of the RNase under study compared with the WT strain(Differentially expressed 5′ and 3′ endswere defined (using edgeR). The 3′-to-5′ exoRNase processing positions are identified as the RNA 3′ ends more abundant in the WT strain (stop positions) or more abundant in the Δ *exornase* strain (start positions).To investigate the interplay between RNase Y and 3'-to-5' exoRNases in RNA degradation, the RNA 3' ends generated by these enzymes were compared. The trimming stop position of 3'-to-5' exoRNases often corresponded to RNA 3' ends produced by RNase Y, suggesting that the trimming start position of the 3'-to-5' exoRNase aligns with the original RNase Y cleavage. **(B)** 5' and 3' end-seq and REND-seq: total RNA is extracted from WT, RNase Y, and RNase J1 deletion mutant (Δ *rny*Δ*rnjA*), RNase J1 deletion mutant (Δ*rnjA*), and quadruple YhaM + RNase PH + PNPase + RNase R deletion mutant (Δ*4exo*). 5’ end sequencing was applied to the Δ*rnjA* and WT strains to uncover the 5' ends produced by endoRNases (RNase Y in this example). An RNA adapter was ligated to RNA 5′ ends to specifically capture the 5′ P ends of RNAs. After fragmentation and size selection, the RNAs were dephosphorylated, polyadenylated and RT using a barcoded primer. cDNAs were amplified with primers containing Illumina adapters and sequenced using single-end sequencing on NextSeq500. 3' end sequencing was applied to WT and Δ*4exo* strains to uncover the 3' ends produced by RNase Y. The 3' end sequencing procedure was performed as in REND-seq (Fig. 6D), with one distinction i.e. omitting the dephosphorylation step prior to the 3' end ligation, to exclude RNA 3' P ends. REND-seq is applied to endoRNase deletion strain (here Δ *rny*Δ*rnjA*) to identify putative cleavage positions as described in Fig. 6D. Analysis of adjacent 5' and 3' ends (peaks in end sequencing), identified in the exoRNase deletion strains by end-sequencing, was used to map putative cleavage sites. The assignment of these sites to a specific endoRNase was accomplished by examining data obtained by 5' end-sequencing and REND-seq analyses performed on the endoRNase deletion strain (i.e. Δ*rny*Δ*rnjA*).

In the methods described above, identification of the processing sites relies on mapping the 5′ ends of RNAs. However, if the cleavage product carrying the newly generated 5′ end is degraded, the RNase processing site will not be identified. To overcome this problem, we have developed a method, named ISCP, that in addition to the RNA 5′ ends, also maps the RNA 3′ ends (Le Rhun et al. 2017) (Fig. 7A represents an updated version of ISCP). ISCP, which consists of comparing the RNA 5′ and 3′ end abundance between a WT and a ∆*rnase* strain (Fig. 7A), has been successively applied to the study of RNase III in *S. pyogenes*. In ISCP, cDNA libraries containing either both primary and processed transcripts or enriched only in processed transcripts were generated and sequenced. The number of reads starting (5′ ends) and ending (3′ reads) at each nucleotide position was calculated not only in the WT and ∆*rnc* strains, but also in additional strains used as further controls. RNase III cleavage sites were identified on the basis of the criterion that the number of read ends resulting from an RNase III processing events is decreased in the Δ*rnc* strain compared with the reference strains. To identify RNase III cleavage sites, RNA ends that were more abundant in the WT strain than in the ∆*rnc* strain were filtered using strict parameters. In total, we identified 92 RNase III cleavage sites, 44 of which had never been identified before with the majority of the positions located in UTRs.

RNase III processing events were classified according to whether the cleavage sites were located in a stem loop or in an antisense duplex. However, for several targets, only one cleavage site was identified, *i.e*. the second position cleaved during the same processing event was not retrieved. ISCP revealed that RNase III often cleaves only one of the two positions, and the importance of this broad nicking activity for transcription regulation is still unknown. ISCP revealed that RNase III preferentially cleaves UTR regions, as has been reported in *S. aureus* (Lioliou et al. 2012). By processing UTRs, RNase III uncouples the expression of genes transcribed within the same RNA molecule.

We found that RNase III has little impact on the metabolism of asRNAs in *S. pyogenes*, in contrast to what has previously been observed in *S. aureus* (Lasa et al. 2011, Lioliou et al. 2012). This limited effect of RNase III on antisense transcription is consistent with what has been described in *B. subtilis* (DiChiara et al. 2016).

#### RNase E

The major endoRNase in Gram-negative bacteria, RNase E, preferentially targets 5′ P RNAs and can catalyze multiple processing events in the same RNA molecule.

Clarke and colleagues provided a genome wide overview of RNase E cleavage sites by mapping the RNA 5′ P ends in *E. coli* (Clarke et al. 2014). In this study RNA-seq was used to determine RNase E targets *in vivo* and *in vitro* by using a thermosensitive RNase E mutant strain and an RNase E variant unable to detect the 5′ P group, respectively. In both cases, the library preparation was performed by capturing the 5′ P end of the RNA by adapter ligation. The number of reads starting at each position were calculated in the different samples to estimate the RNase E processing sites.

For the *in vitro* study, total RNA was extracted and subjected to different enzymatic treatments in order to obtain only 5′ P or 5′ OH RNAs. RNA-seq analysis before and after incubation with RNase E mutated in the 5′ P binding pocket revealed that the recognition of 5′ P is not required for RNase E activity. Several cleavage sites identified *in vivo* by mapping the RNA 5′ P ends in both WT and thermosensitive RNase E mutant, in which RNase E is inactive at nonpermissive temperature, were also detected *in vitro* using RNase E 5′ P binding mutant and target 5′ PPP RNAs. Taken together, these results support a new model of RNase E degradation pathway in which, contrary to what had been postulated, 5′ P detection is not required for target degradation. In this new pathway—named the direct entry pathway—RNase E interacts with the targets via unpaired regions and cleaves them multiple times.

*In vivo* RNA-seq to map RNA 5′ P ends was applied not only to RNase E but also to RNase G (i.e. an RNase E paralogue), revealing novel targets and possibly new functions for this enzyme (Clarke et al. 2014). Inactivation of RNase G led to the accumulation of over 80 5′ P RNA fragments, suggesting a role in the removal of 5′ regions from mRNAs.

Subsequently, transiently inactivating an endoribonuclease followed by RNA-seq (TIER-seq) has been widely used to identify RNase E cleavage sites in various proteobacteria (Chao et al. 2017, Förstner et al. 2018, Hoyos et al. 2020) and in the cyanobacterium *Synechocystis* sp. PCC 6803 (Hoffmann et al. 2021) (Table 2). It was originally developed to study RNase E in *S. typhimurium* (Chao et al. 2017) (Fig. 6B). Since the gene encoding RNase E is essential, Chao and colleagues took advantage of an RNase E thermosensitive mutant strain, as previously done by Clarke et al. RNA-seq was performed in duplicates before and after exposure to nonpermissive temperature (44°C) in the WT and RNase E thermosensitive mutant strains (Fig. 6B). To identify RNase E processing sites, the number of reads per nucleotide of the WT strain grown at a nonpermissive temperature was compared, using DESeq2, to that of the RNase E thermosensitive mutant grown under the same conditions (Fig. 6B). In total, TIER-seq identified 22 033 RNase E cleavage sites, revealing a large amount of new direct targets.

**Figure 8.**
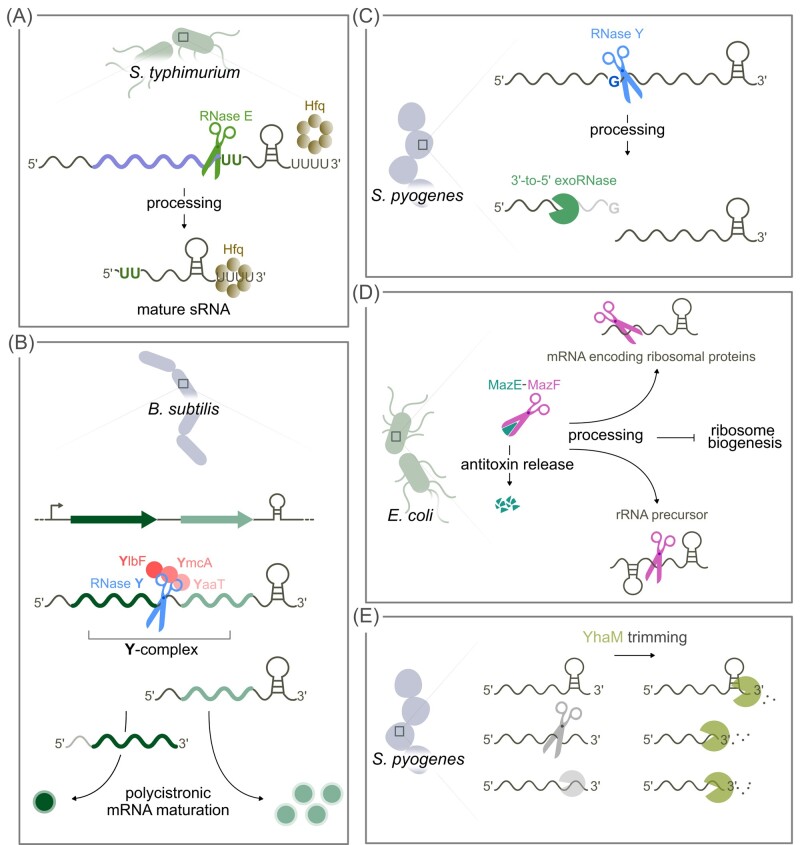
Mapping RNase processing sites reveals new regulatory pathways and the mode of action of RNases. **(A)** In the presence of the chaperon Hfq, RNase E cleaves in proximity to the mRNA stop codons upstream of two uridines. The processing generates functional regulatory sRNAs, which then interact with Hfq. **(B)** RNase Y interacts with YlbF, YmcA, and YaaT to form the Y-complex required for the maturation of polycistronic RNAs, enabling differential regulation of genes encoded within the same operon. **(C)** RNase Y preferentially cleaves target RNAs after a guanosine; upon processing, the fragment upstream of the cleavage site is usually targeted by 3′-to-5′ exoRNases, while the fragments downstream of the processing event are rarely further trimmed. **(D)** The toxin MazF, once freed from the MazE antitoxin, processes mRNA coding for ribosomal proteins and rRNA precursors, impairing ribosome biogenesis, and thus inhibiting translation of its target mRNAs. **(E)** YhaM has a broad trimming activity, removing on average 3 nt from the 3′ ends of RNA after terminators or generated by endoRNA and exoRNase activity.

Alignment of all the RNase E processing sites revealed a minimal consensus motif with a strong preference for a uridine located 2 nt downstream of the processing site. The majority of cleavage sites were located within mRNAs, confirming the key role of RNase E in the initiation of RNA degradation. By analyzing the distribution of the RNase E processing sites, the authors noticed an enrichment in the 3′ region of RNA, close to the mRNA stop codons. RNase E processing in these RNA 3′ UTRs is responsible for the generation of several sRNAs that retain the terminator stem-loop structure from the precursor mRNA, which could interact with the chaperone Hfq (Fig. 8A). Importantly, RNase E processing is not only required for the biogenesis of these sRNAs from 3′ UTRs, but is also required for the sRNA activity.

TIER-seq was also applied to identify RNase E targets in *R. sphaeroides*, a photosynthetic α-proteobacterium in which RNase E has previously been documented to be crucial in the generation of sRNAs (Adnan et al. 2015, Peng et al. 2016). Like in the analysis by Chao *et al*, the authors mapped RNase E cleavages sites by identifying the positions of RNA 5′ ends that were more abundant in the WT strain than in a strain expressing the *E. coli* thermosensitive RNase E (Hoffmann et al. 2021). Alignment of RNase E cleavage site revealed that one uridine and one adenosine are the preferred nucleotides just around the processing position. In contrast to RNase E in *S. typhimurium*, the authors did not find an enrichment of RNase E cleavage sites in proximity to mRNA stop codons, but rather at start codons in the mRNA 5′ UTRs. This study confirms the key role of RNase E in the maturation of sRNAs derived either from UTRs or transcribed from their own promoter. In addition, RNase E was shown to be involved in the processing of several transcripts encoding photosynthetic complexes and regulators of the oxidative stress response.

Finally, TIER-seq was recently applied to identify RNase E cleavage sites in *V. cholerae* (Hoyos et al. 2020). The majority of the processing sites were identified in ORFs and a minimal motif rich in adenosine and uracil was observed, similar to that described in *S. typhimurium*. However, in contrast to the results of TIER-seq in *S. typhimurium*, the RNase E processing sites were not highly enriched in the vicinity of mRNA stop codons. To better understand the role of RNase E in the generation of 3′ UTR-derived sRNAs, the RNA 5′ ends identified by TIER-seq were compared with the 5′ terminal region of known sRNAs. The maturation of several sRNAs was found to be RNase E-dependent. Interestingly, some of these sRNAs interact with the mRNA from which they originate, acting as autoregulatory elements, blocking translation and/or inducing transcription termination.

Overall, while the 3′ end processing of mRNAs is an important mechanism of sRNA biogenesis in Gram-negative bacteria (Ponath et al. 2022), it does not appear to be a widespread strategy in Gram-positive bacteria, where 5′-to-3′ exoRNases likely prevent the accumulation of 3′ UTR-derived fragments (Mediati et al. 2021).

#### RNases J1/J2

RNase J1 is a 5′-to-3′ exoRNase found mainly in Gram-positive bacteria, with the exception of *H. pylori*, which expresses a homolog of RNase J1 (RNase J). While Gram-positive bacteria with high guanosine and cytosine content (high-GC) display one orthologues (RNase J1), bacteria with low-GC content encode two RNase J orthologues (RNase J1 and J2).

The EMOTE protocol was initially developed to identify the direct targets of RNases J1/J2 (Linder et al. 2014, Redder 2015) and subsequently applied to the study of RNase Y in *S. aureus* (Khemici et al. 2015). The library preparation in EMOTE was designed to specifically annotate genome-wide the RNA 5′ P ends (Fig. 6C). By identifying the first base of the mapped sequence, the location of each RNA 5′ P was obtained (Fig. 6C). The EMOTE coverage was calculated at each location as the number of reads whose 5′ end maps at the nucleotide of interest. To identify the RNA 5′ P ends generated by an RNase, EMOTE must be applied to both a WT strain and the deletion mutant of the RNase under study. Comparison of the RNA 5′ P ends in the two strains enabled the identification of the cleaved RNAs and the position of the processing. This analysis revealed an enrichment of RNA 5′ P ends in the RNase J1 or J2 deletion mutant compared to the WT strain. This accumulation is indicative of the 5′-exonucleolytic activity of the RNase J1 and J2 complex *in vivo*. Indeed, in the absence of these enzymes, RNA 5′ P ends produced by RppH or by an endoRNase are no longer degraded and are therefore detectable in the RNase J1 and J2 deletion mutants. To facilitate interpretation of the EMOTE data, the authors also included in the analysis a double RNase J1 and J2 deletion mutant and a strain expressing an inactive variant of RNase J1. The enrichment of RNA 5′ P ends in the presence of an inactive RNase J1 is equal to that observed in the double deletion mutant, indicating that RNase J1 is the main enzyme responsible for the 5′-exonucleolytic degradation and RNase J2 plays more a supportive role for this activity. EMOTE revealed that RNases J1 and J2 are both involved in the maturation of 16S rRNA and the RNA-competent ribonucleoprotein RNase P.

A combination of RNA-seq expression analysis and mapping of RNA 5′ ends was used in *M. tuberculosis* to study the role of RNase J in drug resistance mechanisms (Martini et al. 2022). Construction of libraries containing either all RNA 5′ ends (i.e. primary and processed) or only processed RNA 5′ ends allowed the identification of highly structured decay intermediate fragments accumulating in the ∆*rnj* (Martini et al. 2021, Martini et al. 2022). Through its regulation of RNA degradation, RNase J indirectly influences drug tolerance in *M. tuberculosis*.

#### RNase Y

RNase Y is an ssRNA specific endoRNase that is considered the functional equivalent of RNase E in Gram-positive bacteria.

The EMOTE protocol was also used to map the RNA 5′ P ends generated by RNase Y in *S. aureus*. To identify RNase Y cleavage sites, the RNA 5′ P ends mapped in the WT strain were compared not only with the ∆*rny* strain, but also with another strain expressing an inactive RNase Y variant (Fig. 6C). In total, EMOTE identified 99 RNA 5′ P ends produced by RNase Y in *S. aureus*, and sequence alignment of these positions revealed a preference for a guanosine upstream of the processing sites in adenosine/uracil rich regions. It was predicted that the sequence across the RNase Y processing site was less likely to form structured elements than the surrounding areas.

Notably, EMOTE was also applied to a strain in which RNase Y lacks the transmembrane region, providing further insight into the function of the membrane localization of this enzyme. Although removal of the membrane anchor domain did not affect RNase Y activity (the same 99 processing events were detected by EMOTE), the strain expressing this RNase Y variant showed impaired growth compared with the WT, ∆*rny*, and the strain expressing inactive RNase Y. Given that the same RNase Y cleavage sites were detected by EMOTE when RNase Y was not confined at the membrane, which would indicate uncontrolled RNase Y activity, it is possible that the effect of the deletion of the transmembrane domain on bacterial fitness is unrelated to RNA decay.

DeLoughery and colleagues applied RNA end-enrichment sequencing (REND-seq) to study the involvement of RNase Y in operon maturation in *B. subtilis* by mapping the 5′ ends produced by RNase Y (DeLoughery et al. 2018) (Fig. 6D). REND-seq was initially developed to determine different operon architectures in *B. subtilis*, revealing that the stoichiometry of proteins belonging to the same pathway and/or operon is quantitatively conserved in evolutionarily distant bacterial species (Lalanne et al. 2018). In this study, the authors sought to localize processing events, by mapping RNA 5′ ends, which are key in the maturation of polycistronic transcripts. Consequently, the list of putative RNase Y cleavage sites (approximately 100) was further examined by selecting for processing events resulting in the stabilization of the RNA fragment downstream of the cleavage sites. A total of 22 RNase Y-dependent maturation events, responsible for the production of shorter mRNA isoforms, were identified. REND-seq was applied not only to the WT and ∆*rny* strains, but also to strains deleted from *ylbF, ymcA*, and *yaaT*, which encode proteins capable of interacting with RNase Y and forming the so-called Y-complex (DeLoughery et al. 2016). REND-seq analysis revealed that the Y-complex is required for the majority of the RNase Y maturation events (Fig. 8B) and also affects, together with RNase Y, the abundance of several riboswitches. This study highlighted the determinants of RNase Y processing, proving that the Y-complex is a key factor in the generation of differential RNA isoforms by RNase Y. Interestingly, the comparison of the WT and ∆*ylbF* strains by REND-seq in *S. aureus* revealed that the maturation of operons and processing of two riboswitches also requires YlbF (DeLoughery et al. 2018).

The features and specificity of RNase Y were investigated in *S. pyogenes* by mapping both 5′ and 3′ RNA ends using an optimized and improved ISCP protocol (Fig. 7) (Broglia et al. 2020). A total of 320 RNase Y cleavage sites were identified, including 190 RNA 5′ ends, and 130 RNA 3′ ends (Fig. 7). This analysis revealed a cleavage signature consisting of a guanosine located just upstream of the majority of RNA 5′ ends produced by RNase Y (Fig. 8C), as previously shown in *S. aureus* (Khemici et al. 2015). This is in agreement with our previous data showing that a guanosine was essential for RNase Y processing of the *speB* mRNA, encoding a major virulence factor in *S. pyogenes* (Broglia et al. 2018). Interestingly, ISCP revealed that the fragments upstream and downstream of the RNase Y processing site have different fates. Indeed, while the RNA 3′ ends resulting from the processing (upstream products) are in the majority of the cases targeted by exoRNases, the RNA 5′ ends detected (downstream products) are not further trimmed (Fig. 8C) (see the section ‘Interplay between endo- and exoRNases’).

#### MazF

MazF is a toxin endoRNase belonging to the type II TA system that has been shown to process a variety of RNAs i.e. mRNAs, rRNAs, and tRNAs.

Mapping by overexpression of an RNase in *E. coli* (MORE RNA-seq) has been applied to shed light on the specificity of the MazF toxin endoRNases (Schifano et al. 2014). *Mycobacterium tuberculosis* contains multiple TA systems, including the *mazEF* modules encoding the toxin MazF and the antitoxin MazE. Due to the difficulties associated with studying such systems in *M. tuberculosis*—because of the slow doubling time and limitations of molecular tools—Schifano *et al*. developed an RNA-seq method to characterize the toxin endoRNase of interest (i.e. MazF-mt3) in *E. coli*. The MazF-mt3 is expressed in *E. coli* from a plasmid using a constitutive promoter and, as a control, the same plasmid, without the ORF encoding the toxin, was used. After toxin induction, total RNAs (including rRNAs) were collected and used to prepare two distinct cDNAs libraries, containing either 5′ P or 5′ OH RNAs, respectively.

Application of MORE RNA-seq revealed that MazF-mt3 mainly generates RNA products harboring a 5′ OH group, with the identification of 2 and 273 MazF-mt3-dependent RNA 5′ P and 5′ OH ends, respectively. Alignment of the region surrounding the 273 cleavage sites uncovered a strong consensus sequence consisting of UCCUU, with the processing event occurring after the first uracil. Knowledge of this MazF-mt3 recognition motif was used to deduce MazF-mt3 direct targets in *M. tuberculosis*. MazF-mt3 did not only target mRNAs but also rRNAs, and crucial cleavage sites were identified and validated in the helix/loop 70 of the 23S rRNA and in the anti-Shine-Dalgarno sequence of the 16S rRNA. Based on the MORE RNA-seq results, the authors formulated a model in which MazF would generate specialized ribosomes for the translation of leaderless mRNAs, as has been proposed in *E. coli* (Amitai et al. 2009, Vesper et al. 2011).

The targetome of the toxin endoRNase MazF was also identified in *E. coli* by Culviner and colleagues by using paired-end RNA-seq (Culviner and Laub 2018). RNAs collected from strains expressing and not expressing the MazF toxin were used for cDNA library preparation, and both libraries containing or lacking rRNAs were included in the analysis. RNAs were fragmented, retrotranscribed (using random primers) and end-repaired in order to ligate the adapters to the cDNAs. The deoxyuridine triphosphate (dUTP) method was used to maintain the strand directionality. MazF cleavage sites were identified by comparing the number of reads mapped to each nucleotide in the strain expressing MazF for 5 minutes and the strains in which MazF was not produced at all.

MazF has been shown to cleave the majority of mRNAs expressed in *E. coli*. As previously reported, MazF cleaves ACA sites, but a deeper analysis of the region surrounding the cleavage sites revealed that MazF recognizes a 7nt-long motif containing the ACA site. Given that MazF has previously been described as generating leaderless mRNAs, the authors further explored the RNA-seq data to confirm this observation. Surprisingly, MazF did not generate a pool of leaderless mRNAs, indeed only 10 mRNAs had their leader region shortened by MazF. To determine whether these transcripts were translated more efficiently by ribosomes, as suggested by previous studies, the authors coupled RNA-seq with ribosome profiling. Ribosomal footprints did not increase upon MazF expression, on the contrary, they progressively decreased downstream of the MazF processing event. The authors formulated a model in which extensive MazF processing lead to inhibition of translation of MazF targets. This study is an example of how coupling different omics approaches can provide a more detailed picture of an RNase function. Finally, it has also been reported that MazF generates specialized ribosomes by removal of the anti-Shine-Dalgarno sequence in the 16 rRNA. The authors analysed the cDNA libraries containing rRNAs, but did not identify a processing event that would result in the removal of the anti-Shine-Dalgarno region. Instead, the analysis revealed that MazF alters rRNA biogenesis, interrupting mRNA translation (Fig. 8D).

This method has recently been used to better understand the activity and function of endoRNase toxins (LeRoux et al. 2020). Although TA systems can increase their transcription under stress conditions (Muthuramalingam et al. 2019, Ronneau and Helaine 2019), this study revealed that TA upregulation does not involve toxin activation. The activity of eight endoRNase toxins was monitored by RNA-seq, at different time points after exposure to a stress. The ratio of reads mapped to each nucleotide was calculated in the WT strain, in a strain lacking all TA systems and in strains each overexpressing one of the toxins. Bioinformatic analyses revealed no endoRNase processing events, demonstrating the lack of toxin release.

#### RppH

RppH functions as a decapping enzyme that converts the RNA 5′ PPP end of newly transcribed RNAs into a RNA 5′ P end. This triggers the degradation of the 5′ P RNAs by different types of RNases, whose catalytic activity depends on the recognition of a 5′ P chemical group. Several reports have evaluated the impact of RppH on RNA degradation by using transcriptomics analysis (Supplementary Data 1), (Deana et al. 2008, Bonnin and Bouloc 2015, Frindert et al. 2018). Subsequently, Bischler T. et al. not only identified genes differentially expressed in an *rppH* deletion strain (Δ*rppH*) of *H. pylori* compared with the WT strain, but also adapted the dRNA-seq protocol (Sharma et al. 2010, Sharma and Vogel 2014) to identify direct targets of RppH, i.e. RNAs that are dephosphorylated by this enzyme (Bischler et al. 2017). The phosphorylation status of *H. pylori* was monitored by constructing and sequencing three different cDNA libraries: (i) containing only 5′ PPP RNAs; (ii) containing only 5′ P RNAs; and (iii) containing both 5′ P and 5′ PPP RNAs. These distinct libraries were generated using a combination of enzymatic treatments with TAP and TEX, as described previously (see ‘introduction on RNA-seq’) in the WT, Δ*rppH*, and complemented strains. 63 transcripts (53 mRNAs and 10 sRNAs) RppH direct targets were identified by selecting transcripts that—in the absence of RppH—were more abundant and harbored a 5′ PPP group. Based on the limited number of RppH targets in *H. pylori*, the authors hypothesized the presence of a second, nonredundant pyrophosphohydrolase, as has been proposed in other bacterial species such as *B. subtilis* (Hsieh et al. 2013, Bonnin and Bouloc 2015).

### Interplay between endo- and exoRNases

Sequencing and mapping of the RNA 3′ ends enabled the identification of 3′-to-5′ exoRNase targets, whose processing positions have never been determined genome-wide at nucleotide resolution. The ISCP method was therefore used to identify the direct targets of the 3′-to-5′ exoRNases PNPase, RNase R and YhaM in *S. pyogenes* (Fig. 7A) (Lécrivain et al. 2018). The more abundant RNA 3′ ends in one of the 3′-to-5′ exoRNase deletion strains (∆*exornase*) and the more abundant 3′ ends in the WT strain were defined as the start and stop positions of exoRNase trimming, respectively. The distance between the start and stop positions defines the length of the exoRNase trimming from 3′ to 5′. Knowledge of the position at which an exoRNase starts and eventually stops the degradation of an RNA molecule provided insight into the activity of 3′-to-5′ exoRNase *in vivo*.

We found that YhaM is an unspecific 3′-to-5′ exoRNase that removed an average of 3 nt from the majority of the transcripts (Fig. 8E). The function of this general trimming activity remains unclear and further studies are needed to decipher the importance of this phenomenon. ISCP revealed that PNPase is the major 3′-to-5′ exoRNase in *S. pyogenes*, while RNase R has a limited impact in RNA degradation, at least under the standard growth conditions used in the study (Lécrivain et al. 2018).

Remarkably, knowledge of the exact location of both endoRNase and exoRNase processing sites on a genome-wide scale offers the possibility of studying RNA degradation pathways no longer on a single RNA molecule but as system-level analysis. To this end, an RNA-seq based comparative approach has been developed to uncover the interplay between RNase Y and 3′-to-5′ exoRNases in *S. pyogenes* (Broglia et al. 2020). This analysis makes it possible to compare the targetomes of different RNases by matching the positions corresponding to the RNA ends produced by the RNases under study (Fig. 7A). The comparative analysis revealed that while the RNA 5′ ends produced by RNase Y corresponded to the original RNase Y processing site, the RNA 3′ ends mainly originated from further trimming by 3′-to-5′ exoRNases, following the RNase Y processing event (Fig. 8C). The RNA 3′ ends, more abundant in the WT strain than in the Δ*rny* strain, often do not correspond to the location of RNase Y processing event, but rather to the position at which a 3′-to-5′ exoRNase stopped the trimming after RNase Y activity. The retrieval of the original RNase Y processing event (i.e. the position at which this 3′-to-5′ exoRNase started the trimming) enabled more precise mapping of the RNase Y targetome (Fig. 7A). This comparative approach also simplifies the identification of processing events that would otherwise be undetectable. Consequently, the application of this method enables a more accurate annotation and understanding of the targetome of an endoRNase.

The comparative method revealed that PNPase is the major exoRNase acting in concert with RNase Y in RNA degradation in *S. pyogenes*, removing the RNA fragments resulting from RNase Y activity. The interplay between RNase Y and PNPase is crucial for the maturation of polycistronic transcripts and for the degradation of RNA fragments derived from regulatory regions, e.g. riboswitches.

This approach makes it possible to characterize the interplay of multiple RNases and obtain a global picture of how RNA degradation is carried out in bacteria. The simultaneous identification of different RNase targetomes followed by their comparison is a powerful methodology for characterizing these enzymes *in vivo*. Notably, a similar approach was recently used to dissect the interplay of PNPase with RNases III and RNases E in the facultative phototrophic *R. sphaeroides*. By comparing the RNA 3′ ends produced by these RNases, the authors demonstrated that upon cleavage by RNases E or RNases III, the RNA 3′ ends are further trimmed by PNPase (Spanka et al. 2021).

Massive 3′ end mapping (3′ end-seq) in *E. coli* revealed a high number of 3′ RNA sites within the same transcription unit (Herzel et al. 2022). Comparison of the mapped RNA 3′ ends with RNA 5′ ends produced by RNase E, which were previously identified in (Clarke et al. 2014), revealed that the majority of these internal RNA 3′ sites derive from RNase E processing events. The match between internal RNA 3′ sites and previously annotated RNase E processing events increases when RNA 3′ ends identified in the Δ*pnpA* strain were used in the comparison, suggesting that PNPase is responsible for degrading RNA fragments produced by RNase E (Herzel et al. 2022).

During the preparation of this review, this 3′ end-seq approach has been used together with mapping of the RNA 5′ ends and REND-seq (DeLoughery et al. 2018) to study in detail the interplay between endo- and exoRNase in *B. subtilis* (Taggart et al. 2023) (Fig. 7B). This preprint provides a comprehensive targetome of RNase Y in *B. subtilis*, by mapping RNA 5′ ends in a strain lacking 5′-to-3′ exoRNase activity (*i.e*. RNase J1) and 3′ ends in a strain lacking all four 3′-to-5′ exoRNases (i.e. PNPase, RNase R, RNase PH, and YhaM) (Taggart et al. 2023) (Fig. 7B). The mapping of endoRNase Y cleavage in the absence of exoRNases, which degrade the newly generated RNA fragments, offers the possibility of mapping both RNA ends generated during the same endoribonucleolytic processing event. Identification of RNA end mapping in exoRNase knockout strains is followed by comparison with the results obtained in the strains lacking the endoRNase under study (Fig. 7B). This study refined the cleavage signature of RNase Y in *B. subtilis*, consisting of a purine (preferentially a G) and an adenosine downstream and upstream of the processing site, respectively, within a region characterized by low GC content. In addition, a preference for a structured RNA was detected downstream of the processing site. Bioinformatic analysis of the RNA ends detected in all the strains tested revealed the presence in *B. subtilis* of a yet unidentified RNase capable of trimming both 5′ P and 5′ OH RNAs (Taggart et al. 2023).

## Conclusions and outlook

Advances in RNA-seq based methods for studying bacterial RNase unraveled many unknowns concerning the activity and functions of these enzymes. However, these methods have intrinsic limitations that researchers must take into account when designing an RNA-seq experiment for RNase target identification. The methods for mapping RNA ends produced by endoRNase require that at least the RNA fragment upstream or downstream of the cleavage site is sufficiently stable to be detected by RNA-seq. However, it is highly likely that, following the processing event, both RNA products are rapidly degraded and therefore not detectable. In this case, the RNase target could be deduced from differentially expression analysis, as the transcript would be more abundant in the WT strain than in the strain lacking the RNase under study. It should be noted, however, that complete degradation of an RNA molecule does not always lead to a change in its abundance. Indeed, feedback loops in regulatory networks may overcome the possible variations in gene expression.

A strategy to ensure the detection of RNA products resulting from endoRNase cleavages is to include many control strains in the analysis, such as exoRNase deletion strains and double deletion strain of the endoRNase and exoRNase(s). This would would uncover processing events that would not otherwise be possible to map, thereby refining the endoRNase targetome (Broglia et al. 2020). For instance, when mapping RNA 5′ ends generated by endoRNases in bacterial species harboring 5′-to-3′ exoRNases, it is recommended to perform the annotation also in a strain lacking both the endoRNase and 5′-to-3′ exoRNases. In such a strain, the products upstream of the endoRNase processing event would be detectable, facilitating mapping of the RNA 5′ ends (DiChiara et al. 2016, Taggart et al. 2023).

Another aspect to consider is the ability of RNases to act in an interchangeable manner, *i.e*. when the gene encoding a certain RNase is deleted, another RNase (or RNases) could functionally replace the missing RNase. This characteristic makes the screening of RNase cleavage sites more challenging, as some of the targets would not be detected as such. In this scenario, the inclusion in the study of strains in which other RNases are deleted in addition the one under study could facilitate the discovery of a greater number of processing events. Noteworthy, deletion of one RNase can have effects on the expression of other RNases (Trinquier et al. 2020), so it is possible that some of the cleavage sites detected are not directly due to the RNase under study. Finally, some RNases are essential for bacterial viability, so deletion of these genes is not possible. Researchers have developed various strategies to study essential RNases (e.g. thermosensitive mutants and conditional promoters), and improvements in bacterial genetic tools will certainly facilitate the study of these essential RNases, especially in poorly characterized bacteria. In a recent study, RNA degradation pathways were analysed in 96 culturable and nonculturable species, including complex fecal cultures, paving the way for future research in bacterial RNA metabolism (Huch et al. 2023).

The methods described above provide a list of putative direct RNase targets, and a careful interpretation of these data is essential to select these targets for further investigation. An important criterion to consider is the percentage of cleaved transcripts; indeed, a processing event may occur in the majority of the transcripts or only in a few copies. It is intuitive that biologically relevant processing events are those that affect a considerable number of transcripts. The question remains of how to interpret and study the processing events occurring in only a few RNA molecules. Furthermore, calculating a cleavage ratio is a rather challenging procedure, as upon cleavage, the RNA products may undergo immediate degradation and become undetectable. In this case, the cleavage ratio would only be representative of the detectable RNA fraction.

The next challenges in the field will be, first of all, to study RNases not as single entities, but rather to investigate the interplay of different RNases on a global scale in order to obtain a more integrated view of the regulation of gene expression by RNases. It will also be crucial to exploit the methods described in this review to shed light on RNase activity not only under standard laboratory conditions, as has already been widely done, but also, for instance, under stress and infection conditions. In light of the observation that several RNases play a key role in the regulation of virulence, it is very important to investigate their targets in an infection model. Studying changes in the bacterial transcriptome during the course of an infection, could reveal novel functions for RNases and regulatory sRNAs (Westermann and Vogel 2021). For instance, Dual RNA-seq revealed the function of several sRNAs (e.g. PinT) that would have remained unknown if they had not been studied in the context of host-pathogen interactions (Westermann et al. 2016). We foresee, in the event of changing environmental conditions, a significant remodeling of RNase targetomes, which awaits further investigation. Recent studies on the identification of RNase targets using antibiotic-resistant strains or under infection-like conditions have revealed novel bacterial regulatory mechanisms, paving the way for new drug pathways to combat antibiotic resistance (McKellar et al. 2022, Mediati et al. 2022). Finally, by integrating multiple omics approaches, researchers will gain a better understanding of bacterial RNase activity. For instance, coupling RNA-seq with proteomic analysis and/or ribosome profiling will provide a more comprehensive picture of RNase targets and a better understanding of the effects of a processing event on translation. Recently, sequencing of RNA degradation intermediates harboring an 5′ P RNA (5′ degradome) has emerged as a valuable approach for obtaining a in-depth picture of ribosome positioning along mRNAs under various stress conditions and antibiotic treatments (Huch et al. 2023).

The study of bacterial RNases will undoubtedly benefit from the latest innovations in RNA-seq technologies. The past years have witnessed the development of the first protocols for bacterial single-cell RNA-seq (Blattman et al. 2020, Imdahl et al. 2020, Kuchina et al. 2021), which enable exploration of the gene expression heterogeneity within a bacterial population. The transcriptomic snapshot obtained by standard RNA-seq methods is an overview of the bulk population and is unable to provide information on the diversity of gene expression in single bacterial cells. Similarly, when using standard RNA-seq methodologies to identify the direct targets of RNases, we have identified processing events at the population level, and in general, we assume that the targetome of an RNase is the same in every cell. However, RNases may cleave certain targets only in small subpopulation, potentially remodeling the expression programs controlling important processes such as stress response and antibiotic resistance. In the future, single-cell RNA-seq analysis in bacteria could provide a better understanding of the role of RNases in gene expression heterogeneity within bacterial populations.

The methods for identifying RNase cleavage sites discussed in this review are based on short-read sequencing. This means that RNA ends are not directly annotated from the sequencing of the whole RNA but are identified after that each individual cDNA fragment has been mapped to assemble the full-length RNA molecule. Other sequencing strategies, also named third-generation sequencing technologies [e.g. nanopore sequencing and single-molecule real-time (SMRT) sequencing], enable longer reads to be generated and thus full-length transcripts to be sequenced (Lu et al. 2016, Amarasinghe et al. 2020). This enables larger transcriptional units and RNA termini to be mapped more precisely. For instance, the nanopore technology has recently been used in prokaryotes and has shed light on the mechanism of rRNA processing in archaea (Grünberger et al. 2022). Full-length primary transcripts were sequenced in *E. coli* using SMRT-Cappable-seq, deciphering the complexity of operon structure (Yan et al. 2018). SMRT-Cappable-seq revealed extensive read-through at the transcription termination sites demonstrating that this phenomenon is a regulated process that controls the elongation of transcriptional unit. The ability to precisely map the RNA 5′ and 3′ ends by sequencing both full-length primary and processed transcripts will facilitate the identification of processing events in an unbiased and accurate manner.

Several factors control RNA accessibility and recognition by RNases, including interacting molecules, RNA structure, RNA modifications, and translation. Most of these features have been studied for individual target RNAs. Little is known, on a genome-wide scale, about the impact of RNA structure or modifications on RNase activity. Global profiling of RNA structure *in vitro* by parallel analysis of the mRNA structure (PARS) in combination with ribosome profiling and standard RNA-seq revealed the structural signature of RNase E in *E. coli* (Del Campo 2015 Plos Genetics). 2'-hydroxyl acylation analysed by primer extension and mutational profiling (SHAPE-MaP) coupled with RNA-seq was used to identify the *E. coli* structurome at the transcriptome scale (Mustoe et al. 2018). This study highlights many structural motifs occurring in UTRs or intergenic regions that correspond to RBP-binding or RNase processing sites, suggesting that RNA structure plays a key role in the post-transcriptional regulation of gene expression. High-throughput structure probing experiments will not only unveil the structural preferences of RNases, but also elucidate how the structurome changes upon RNase processing and its functional consequences.

While our understanding of RNA modifications derives mainly from studies in eukaryotes, a growing body of research in bacteria has revealed that modified ribonucleotides are not limited to tRNAs and rRNAs, but are also present in regulatory RNAs and mRNAs (Marbaniang and Vogel 2016; Antoine *et al*. 2021). RNA-seq-based approaches have been developed to map RNA modifications such as N6-Methyladenosine (m^6^A) (Dominissini et al. 2012, Meyer et al. 2012, Deng et al. 2015) and Nicotinamide-Adenine Dinucleotide (NAD) at the RNA 5′ ends (Cahová et al. 2015). The MazF RNase is unable to cleave the ‘ACA’ motifs when modified to ‘m^6^A-CA’; of note, this characteristic has been harnessed for the precise mapping of m^6^A sites (Imanishi et al. 2017, Garcia-Campos et al. 2019). The NAD modification at the RNA 5′ ends can protect RNAs from processing and degradation by RppH and RNase J1/J2 in Gram-negatives and Gram-positives, respectively (Cahová et al. 2015, Frindert et al. 2018). We envision that RNA modifications have a major effect on RNase activity by masking processing sites or possibly by recruiting these enzymes and other RBPs. Undoubtably, combining the mapping of RNase processing events with the transcriptome-wide identification of RNA modifications will offer a comprehensive understanding on the intricate regulation of RNase activity and RNA metabolism in bacteria.

Ribosomes trafficking along mRNAs has a major impact on RNase activity and therefore RNA stability. This year, an atlas of the mRNA degradome across the bacterial tree of life, based on sequencing of 5′ P degradation products, revealed that cotranslational RNA degradation is widespread and conserved in both Gram-negative and Gram-positive bacteria (Huch et al. 2023). Microorganisms expressing the 5′-to-3′ exoRNase J1 exhibit a periodicity of 5' P counts occurring every 3 nucleotides. This observation suggests that, similar to the eukaryotic 5′-to-3′ exoRNAse XRN1, exoRNase J1 trails the last ribosome engaged in the mRNA translation. In species lacking a 5′-to-3′ exoRNase, the 3 nt periodicity was less prominent; instead, an accumulation of 5' P endonucleolytic sites in proximity to start and stop codons was observed (Huch et al. 2023). Alterations in ribosome positioning resulting from changing environmental conditions, e.g. antibiotic treatment, have an impact on RNA degradation patterns. This study provides an exemplary demonstration of how in-depth information on bacterial post-transcriptional regulation can be obtained using RNA-seq mapping of processed RNA 5' P ends.

Overall, RNA-seq methodologies have revolutionized the field of RNA biology in bacteria, and the development of novel strategies will continue to reveal new insights into the functions of RNases as key players in the post-transcriptional mechanism of gene expression regulation.

## Supplementary Material

fuad049_Supplemental_File
