# Supplementary material for: Methodologies for bacterial ribonuclease characterization using RNA-seq
Source: FEMS Microbiol Rev. 2023 Sep 1;47(5):fuad049. doi: 10.1093/femsre/fuad049 (PMC10503654; doi:10.1093/femsre/fuad049)
Supplement: fuad049_Supplemental_File [file fuad049_supplemental_file.pdf]

Supplementary Data for

Methodologies for bacterial ribonuclease characterization using RNA-seq

Laura Broglia[1,2], Anaïs Le Rhun[1,3*] and Emmanuelle Charpentier[1,4*]

[1]Max Planck Unit for the Science of Pathogens, D-10117 Berlin, Germany

[2]Center for Human Technologies, Istituto Italiano di Tecnologia, 16152 Genova, Italy

[3]Univ. Bordeaux, CNRS, INSERM, ARNA, UMR 5320, U1212, F-33000 Bordeaux, France

[4]Institute for Biology, Humboldt University, D-10115 Berlin, Germany

*To whom correspondence should be addressed.

Correspondence:

Emmanuelle Charpentier; Tel: +493028460410; Email: research-charpentier@mpusp.mpg.de

Anaïs Le Rhun; Tel: +33 55 7574 565; Email: anais.le-rhun@inserm.fr

This PDF file includes Supplementary Data 1

Transcriptomic studies					
**Method**[a]	**RNase**	**Mechanism of action**[b]	**Organisms**	**Selected main findings**	**References**
Microarray / Microarray after rifampicin treatment	RNase III	dsRNA endoRNase	*E. coli*	RNase III directly controls the biofilm-dependent modulation protein	(Sim *et al.* 2010)
		dsRNA endoRNase	*S. coelicolor*	RNase III affects genes involved in secondary metabolism, differentiation and response to stress	(Huang *et al.* 2005)
	RNase E	ssRNA endoRNase	*E. coli*	RNase G overexpression allows cell viability in the absence of RNase E	(Lee, Bernstein and Cohen 2002)
				The degradosome is not required for the degradation of all the transcripts	(Bernstein *et al.* 2004)
	RNase G	ssRNA endoRNase	*E. coli*	RNase G controls the abundance of 11 mRNAs (involved in the utilization of energy sources)	(Lee, Bernstein and Cohen 2002)
	RNase Y	ssRNA endoRNase	*S. aureus*	RNase Y is involved in virulence gene regulation and controls the expression of 20% of genes	(Marincola *et al.* 2012)
					(Numata *et al.* 2014)
			S. pyogenes	RNase Y regulates virulence genes according to the bacterial nutritional status	(Kang, Caparon and Cho 2010)
			B. subtilis	RNase Y depletion affects the expression of 900 transcripts including mRNAs involved in biofilm formation	(Lehnik-Habrink *et al.* 2011)
			T. thermophilus	RNase Y is not the major endoRNase in *T. thermophilus*	(Ohyama *et al.* 2014)
			C. perfringens	RNase Y affects the expression toxin-producing genes	(Obana, Nakamura and Nomura 2017)
	YbeY	ssRNA endoRNase	*T. thermophilus*	YbeY is involved in mRNA degradation	(Ohyama *et al.* 2014)
			S. meliloti	Hfq-dependent sRNA-mediated silencing of amino acid transport requires YbeY	(Saramago *et al.* 2017)
	PNPase	3′-to-5′ exoRNase	*E. coli*	PNPase controls the steady-state level of higher number of mRNAs than RNase II	(Mohanty and Kushner 2003)
				Decay of some transcripts is performed by the single components of the degradosome	(Bernstein *et al.* 2004)
				In the absence of PNPase, 200 mRNAs belonging to the carbon metabolism are stabilized.	(Dressaire *et al.* 2018)
			T. thermophilus	PNPase has a major role during stationary phase than exponential growth phase	(Ohyama *et al.* 2014)
	RNase R	3′-to-5′ exoRNase	*E. coli*	RNase R does not affect the overall stability of mRNAs	(Dressaire *et al.* 2018)
	RNase II	3′-to-5′ exoRNase	*T. thermophilus*	RNase II gene had no significant effect on global mRNA abundance	(Ohyama *et al.* 2014)

			E. coli	RNase II protects mRNAs from degradation by other RNases	(Mohanty and Kushner 2003)
	RNase J1/J2	5′-to-3′ exoRNase/ (RNase J2 also endoRNase)	*B. subtilis*	RNase J1 depletion affects 79 transcripts and have overlapping substrates with RNase J2	(Mäder *et al.* 2008)
					(Mäder *et al.* 2008)
			E. faecalis	RNase J2 controls genes involved in virulence and fitness	(Gao *et al.* 2017)
	RppH	pyrophosphohydrolase	*E. coli*	RppH promotes RNA degradation	(Deana, Celesnik and Belasco 2008)
				The rate of RppH-dependent RNA degradation is limited either by pyrophosphate removal or by subsequent RNase E cleavage	(Luciano *et al.* 2012)
	RNase Y	ssRNA endoRNase	*S. pyogenes*	RNase Y deletion causes a 2-fold increase in RNA stability	(Chen *et al.* 2013)
Tiling array	RNase III	dsRNA endoRNase	*B. subtilis*	RNase III has a minor role in RNA degradation	(Durand *et al.* 2012)
			E. coli	RNase III controls 12% of the coding mRNAs	(Stead *et al.* 2011)
	RNase E	ssRNA endoRNase	*E. coli*	RNase E affects the abundance of 60% of the transcripts	(Stead *et al.* 2011)
	RNase Y	ssRNA endoRNase	*B. subtilis*	RNase Y depletion affects 26% of genes and 51% of protein coding genes	(Durand *et al.* 2012)
					(Laalami *et al.* 2013)
	RNase J1/J2	5′-to-3′ exoRNase/ (+ endoRNase for J2?)	*B. subtilis*	RNase J1 depletion affects 30% of the genome	(Durand *et al.* 2012)
RNA-seq	RNase III	dsRNA endoRNase	*S. aureus*	RNase III digests overlapping transcripts	(Lasa *et al.* 2011)
			S. coelicolor	RNase III regulates genes with regulatory or metabolic functions	(Gatewood *et al.* 2012)
			S. pyogenes	RNase III has a limited impact both on antisense transcripts and on global gene	(Le Rhun *et al.* 2017)
			B. pertussis	RNase III controls the expression of ~25% of the transcriptome	(Ifill *et al.* 2021)
	RNase E	ssRNA endoRNase	*C. Synechocystis*	RNase E alters the expression of larger number of chromosomal genes and antisense RNAs	(Cavaiuolo *et al.* 2020)
			M. tuberculosis	RNase E plays an important pleiotropic role in mycobacterial RNA metabolism	(Płociński *et al.* 2019)
			B. pertussis	RNase E controls the expression of ~25% of the transcriptome	(Ifill *et al.* 2021)
	RNase Y	ssRNA endoRNase	*S. pyogenes*	RNase Y processes 15 operons	(Chen *et al.* 2018)
			B. subtilis	The Y-complex (RNase Y, YlbF, YmcA, and YaaT) has an effect on the stability of most riboswitches and RNA leader sequences	(DeLoughery *et al.* 2018)
			S. pyogenes	RNase Y affects 4.4% of the transcriptome	(Broglia *et al.* 2020)

		S. pneumoniae	RNase has key role in pneumococcal physiology	(Sinha *et al.* 2021)
YbeY	ssRNA endoRNase	*Y. enterolitica*	YbeY is involved in 16S rRNA maturation and controls the expression of virulence genes	(Leskinen, Varjosalo and Skurnik 2015)
PNPase	3′-to-5′ exoRNase	*E. coli*	PNPase affects the levels of stable RNAs and controls the expression of genes involved in motility and biofilm formation	(Pobre and Arraiano 2015)
			First transcriptomic study comparing the roles of exoRNases in the exponential to stationary phase transition	(Pobre *et al.* 2019)
			PNPase degrades RNA fragments derived from mRNAs targeted by sRNAs	(Cameron *et al.* 2019)
		B. subtilis	PNPase is the major 3′-to-5′ exoRNase to degrade decay intermediate fragments.	(Liu *et al.* 2014)
		P. aeruginosa	PNPase is required for the T3SS and bacterial virulence	(Chen *et al.* 2016)
		S. pyogenes	PNPase is the major 3′-to-5′ exoRNase degrading endoRNase fragments	(Lécrivain *et al.* 2018)
		M. tuberculosis	PNPase controls the levels of non-coding RNA species (tmRNA, *rnpB* and a 6S-like molecule)	(Płociński *et al.* 2019)
		S. pneumoniae	PNPase controls virulence through sRNA regulation	(Sinha *et al.* 2021)
		R. sphaeroides	PNPase control the abundance of sRNAs and stable RNAs (tRNA and rRNA)	(Spanka, Reuscher and Klug 2021)
RNase R	3′-to-5′ exoRNase	*E. coli*	RNase R controls the expression of genes involved in motility and biofilm formation	(Pobre and Arraiano 2015)
			First transcriptomic study comparing the roles of exoRNases in the exponential to stationary phase transition	(Pobre *et al.* 2019)
			RNase R facilitates mRNA degradation during acclimation phase	(Zhang *et al.* 2018)
		S. pyogenes	RNase R has a limited role in RNA degradation under standard growth conditions	(Lécrivain *et al.* 2018)
		T. thermophilus	RNase R has a major role during stationary phase than exponential growth phase	(Ohyama *et al.* 2014)
RNase II	3′-to-5′ exoRNase	*E. coli*	RNase II controls the expression of genes involved in motility and biofilm formation	(Pobre and Arraiano 2015)
			First transcriptomic study comparing the roles of exoRNases in the exponential to stationary phase transition	(Pobre *et al.* 2019)
YhaM	3′-to-5′ exoRNase	*S. pyogenes*	YhaM targets the majority of RNA ends but does not affect global RNA abundance	(Lécrivain *et al.* 2018)

	RNase J	5′-to-3′ exoRNase	*H. pylori*	RNase J is a major RNase involved in the degradation of the majority of the transcripts in the cell	(Redko *et al.* 2016)
			R. sphaeroides	Deletion of RNase J causes accumulation of RNA fragments	(Rische-Grahl *et al.* 2014)
			M. tuberculosis	RNase J indirectly affects drug tolerance	(Martini *et al.* 2022)
			C. synechocystis	RNase J depletion affects the endogenous plasmid encoded transcripts	(Cavaiuolo *et al.* 2020)
	RNase P	ssRNA endoRNase	*E. coli*	RNase P inactivation affects the abundances of ~46% of the expressed transcripts in *E. coli*	(Mohanty and Kushner 2022)
	RppH	pyrophosphohydrolase	*H. pylori*	Absence of RppH causes the increase in abundance of 179 RNAs	(Bischler *et al.* 2017)
			B. subtilis	RppH affects the expression of 13% of the transcripts	(Frindert *et al.* 2018)
RNA-seq after rifampicin treatment	RNase G	ssRNA endoRNase	*S. maltophilia*	RNase G deletion causes an overall increase in RNA half-life	(Bernardini and Martínez 2017)
	RNase Y	ssRNA endoRNase	*S. aureus*	RNase Y affects both steady-state and half-life levels of 50 ORFs encoding ribosomal proteins, housekeeping genes, chaperones and virulence regulators	(Khemici *et al.* 2015)

Supplementary Data 1. Transcriptomic studies for the identification of RNase targets
[a]RNA-seq, RNA sequencing. [b]dsRNA endoRNase, double-stranded specific endoribonuclease; ssRNA endoRNase, single-stranded specific endoribonuclease; 3′-to-5′ exoRNase, 3′-to-5′ exoribonuclease; 5′-to-3′ exoRNase, 5′-to-3′ exoribonuclease.

References

Bernardini A, Martínez JL. Genome-wide analysis shows that RNase G plays a global role in the stability of mRNAs in *Stenotrophomonas maltophilia*. *Sci Rep* 2017;**7**:16016.

Bernstein JA, Lin P-H, Cohen SN *et al.* Global analysis of *Escherichia coli* RNA degradosome function using DNA microarrays. *Proc Natl Acad Sci USA* 2004;**101**:2758–63.

Bischler T, Hsieh P, Resch M *et al.* Identification of the RNA Pyrophosphohydrolase RppH of *Helicobacter pylori* and Global Analysis of Its RNA Targets. *J Biol Chem* 2017;**292**:1934–50.

Broglia L, Lécrivain A-L, Renault TT *et al.* An RNA-seq based comparative approach reveals the transcriptome-wide interplay between 3′-to-5′ exoRNases and RNase Y. *Nature Commun* 2020;**11**:1–12.

Cameron TA, Matz LM, Sinha D *et al.* Polynucleotide phosphorylase promotes the stability and function of Hfq-binding sRNAs by degrading target mRNA-derived fragments. *Nucleic Acids Res* 2019;**47**:8821–37.

Cavaiuolo M, Chagneau C, Laalami S *et al.* Impact of RNase E and RNase J on Global mRNA Metabolism in the *Cyanobacterium Synechocystis* PCC6803. *Front Microbiol* 2020;**11**, DOI: 10.3389/fmicb.2020.01055.

Chen R, Weng Y, Zhu F *et al.* Polynucleotide phosphorylase regulates multiple virulence factors and the stabilities of small RNAs RsmY/Z in *Pseudomonas aeruginosa*. *Front Microbiol* 2016;**7**:247.

Chen Z, Itzek A, Malke H *et al.* Multiple roles of RNase Y in *Streptococcus pyogenes* mRNA processing and degradation. *J Bacteriol* 2013;**195**:2585–94.

Chen Z, Raghavan R, Qi F *et al.* Genome-wide screening of potential RNase Y-processed mRNAs in the M49 serotype *Streptococcus pyogenes* NZ131. *Microbiology open* 2018;**8**:e00671.

Deana A, Celesnik H, Belasco JG. The bacterial enzyme RppH triggers messenger RNA degradation by 5' pyrophosphate removal. *Nature* 2008;**451**:355–8.

DeLoughery A, Lalanne J-B, Losick R *et al.* Maturation of polycistronic mRNAs by the endoribonuclease RNase Y and its associated Y-complex in *Bacillus subtilis*. *Proc Natl Acad Sci USA* 2018;**115**:E5585–94.

Dressaire C, Pobre V, Laguerre S *et al.* PNPase is involved in the coordination of mRNA degradation and expression in stationary phase cells of *Escherichia coli*. *BMC Genomics* 2018;**19**:848.

Durand S, Gilet L, Bessières P *et al.* Three essential ribonucleases—RNase Y, J1, and III—control the abundance of a majority of *Bacillus subtilis* mRNAs. *PLoS Genet* 2012;**8**:e1002520.

Frindert J, Zhang Y, Nübel G *et al.* Identification, biosynthesis, and decapping of NAD-capped RNAs in *B. subtilis*. *Cell Reports* 2018;**24**:1890-1901.e8.

Gao P, Pinkston KL, Bourgogne A *et al.* Functional studies of *E. faecalis* RNase J2 and its role in virulence and fitness. *PLOS ONE* 2017;**12**:e0175212.

Gatewood ML, Bralley P, Weil MR *et al.* RNA-Seq and RNA immunoprecipitation analyses of the transcriptome of *Streptomyces coelicolor* identify substrates for RNase III. *J Bacteriol* 2012;**194**:2228–37.
Huang J, Shi J, Molle V *et al.* Cross-regulation among disparate antibiotic biosynthetic pathways of *Streptomyces coelicolor*. *Mol Microbiol* 2005;**58**:1276–87.
Ifill G, Blimkie T, Lee AH-Y *et al.* RNase III and RNase E influence posttranscriptional regulatory networks involved in virulence factor production, metabolism, and regulatory RNA processing in *Bordetella pertussis*. *mSphere* 2021;**6**:e00650-21.
Kang SO, Caparon MG, Cho KH. Virulence gene regulation by CvfA, a putative RNase: the CvfA-Enolase complex in *Streptococcus pyogenes*links nutritional stress, growth-phase control, and virulence gene expression. *Infect Immun* 2010;**78**:2754–67.
Khemici V, Prados J, Linder P *et al.* Decay-initiating endoribonucleolytic cleavage by RNase Y is kept under tight control via sequence preference and sub-cellular localisation. *PLoS Genet* 2015;**11**:e1005577.
Laalami S, Bessières P, Rocca A *et al.* *Bacillus subtilis* RNase Y activity *in vivo* analysed by tiling microarrays. *PLoS ONE* 2013;**8**:e54062.
Lasa I, Toledo-Arana A, Dobin A *et al.* Genome-wide antisense transcription drives mRNA processing in bacteria. *Proc Natl Acad Sci USA* 2011;**108**:20172–7.
Le Rhun A, Lécrivain A-L, Reimegård J *et al.* Identification of endoribonuclease specific cleavage positions reveals novel targets of RNase III in *Streptococcus pyogenes*. *Nucleic Acids Res* 2017;**45**:2329–40.
Lécrivain A-L, Le Rhun A, Renault TT *et al.* *In vivo* 3′-to-5′ exoribonuclease targetomes of *Streptococcus pyogenes*. *Proc Natl Acad Sci USA* 2018;**115**:11814–9.
Lee K, Bernstein JA, Cohen SN. RNase G complementation of *rne* null mutation identifies functional interrelationships with RNase E in *Escherichia coli*. *Mol Microbiol* 2002;**43**:1445–56.
Lehnik-Habrink M, Schaffer M, Mäder U *et al.* RNA processing in *Bacillus subtilis*: identification of targets of the essential RNase Y. *Mol Microbiol* 2011;**81**:1459–73.
Leskinen K, Varjosalo M, Skurnik M. Absence of YbeY RNase compromises the growth and enhances the virulence plasmid gene expression of *Yersinia enterocolitica* O:3. *Microbiology (Reading, Engl)* 2015;**161**:285–99.
Liu B, Deikus G, Bree A *et al.* Global analysis of mRNA decay intermediates in *Bacillus subtilis* wild-type and polynucleotide phosphorylase-deletion strains. *Mol Microbiol* 2014;**94**:41–55.
Luciano DJ, Hui MP, Deana A *et al.* Differential control of the rate of 5′-end-dependent mRNA degradation in *Escherichia coli*. *J Bacteriol* 2012;**194**:6233–9.
Mäder U, Zig L, Kretschmer J *et al.* mRNA processing by RNases J1 and J2 affects *Bacillus subtilis* gene expression on a global scale. *Mol Microbiol* 2008;**70**:183–96.
Marincola G, Schäfer T, Behler J *et al.* RNase Y of *Staphylococcus aureus* and its role in the activation of virulence genes. *Mol Microbiol* 2012;**85**:817–32.
Martini MC, Hicks ND, Xiao J *et al.* Loss of RNase J leads to multi-drug tolerance and accumulation of highly structured mRNA fragments in *Mycobacterium tuberculosis*. *PLoS Pathog* 2022;**18**:e1010705.
Mohanty BK, Kushner SR. Genomic analysis in *Escherichia coli* demonstrates differential roles for polynucleotide phosphorylase and RNase II in mRNA abundance and decay. *Mol Microbiol* 2003;**50**:645–58.
Mohanty BK, Kushner SR. Inactivation of RNase P in *Escherichia coli* significantly changes post-transcriptional RNA metabolism. *Mol Microbiol* 2022;**117**:121–42.
Numata S, Nagata M, Mao H *et al.* CvfA protein and polynucleotide phosphorylase act in an opposing manner to regulate *Staphylococcus aureus* virulence. *J Biol Chem* 2014;**289**:8420–31.
Obana N, Nakamura K, Nomura N. Role of RNase Y in *Clostridium perfringens* mRNA decay and processing. *J Bacteriol* 2017;**199**:e00703-16.
Ohyama H, Sakai T, Agari Y *et al.* The role of ribonucleases in regulating global mRNA levels in the model organism *Thermus thermophilus* HB8. *BMC Genomics* 2014;**15**:386.
Płociński P, Macios M, Houghton J *et al.* Proteomic and transcriptomic experiments reveal an essential role of RNA degradosome complexes in shaping the transcriptome of *Mycobacterium tuberculosis*. *Nucleic Acids Res* 2019;**47**:5892–905.
Pobre V, Arraiano CM. Next generation sequencing analysis reveals that the ribonucleases RNase II, RNase R and PNPase affect bacterial motility and biofilm formation in *E. coli*. *BMC Genomics* 2015;**16**:72.
Pobre V, Barahona S, Dobrzanski T *et al.* Defining the impact of exoribonucleases in the shift between exponential and stationary phases. *Sci Rep* 2019;**9**:16271.
Redko Y, Galtier E, Arnion H *et al.* RNase J depletion leads to massive changes in mRNA abundance in *Helicobacter pylori*. *RNA Biol* 2016;**13**:243–53.
Rische-Grahl T, Weber L, Remes B *et al.* RNase J is required for processing of a small number of RNAs in *Rhodobacter sphaeroides*. *RNA Biol* 2014;**11**:855–64.
Saramago M, Peregrina A, Robledo M *et al.* *Sinorhizobium meliloti* YbeY is an endoribonuclease with unprecedented catalytic features, acting as silencing enzyme in riboregulation. *Nucleic Acids Res* 2017;**45**:1371–91.
Sim S-H, Yeom J-H, Shin C *et al.* *Escherichia coli* ribonuclease III activity is downregulated by osmotic stress: consequences for the degradation of bdm mRNA in biofilm formation. *Mol Microbiol* 2010;**75**:413–25.
Sinha D, Frick JP, Clemons K *et al.* Pivotal Roles for Ribonucleases in *Streptococcus pneumoniae* Pathogenesis. *mBio* 2021;**12**:e02385-21.
Spanka D-T, Reuscher CM, Klug G. Impact of PNPase on the transcriptome of *Rhodobacter sphaeroides* and its cooperation with RNase III and RNase E. *BMC Genomics* 2021;**22**:106.
Stead MB, Marshburn S, Mohanty BK *et al.* Analysis of *Escherichia coli* RNase E and RNase III activity *in vivo* using tiling microarrays. *Nucleic Acids Res* 2011;**39**:3188–203.
Zhang Y, Burkhardt DH, Rouskin S *et al.* A Stress response that monitors and regulates mRNA structure is central to cold shock adaptation. *Mol Cell* 2018;**70**:274-286.e7.
